# A Review of OBD-II-Based Machine Learning Applications for Sustainable, Efficient, Secure, and Safe Vehicle Driving

**DOI:** 10.3390/s25134057

**Published:** 2025-06-29

**Authors:** Emmanouel T. Michailidis, Antigoni Panagiotopoulou, Andreas Papadakis

**Affiliations:** 1 Department of Digital Systems, School of Information and Communication Technologies, University of Piraeus, GR18534 Piraeus, Greece; 2 Department of Electrical and Electronics Engineering Educators, School of Pedagogical and Technological Education, GR15122 Athens, Greece; apanagiotopoulou@aspete.gr (A.P.); apapadakis@aspete.gr (A.P.)

**Keywords:** anomaly detection, cybersecurity, deep learning, DL, driver behavior analysis, driving support, emission control, fuel optimization, road perception, machine learning, ML, On-Board Diagnostics II, OBD-II

## Abstract

The On-Board Diagnostics II (OBD-II) system, driven by a wide range of embedded sensors, has revolutionized the automotive industry by enabling real-time monitoring of key vehicle parameters such as engine load, vehicle speed, throttle position, and diagnostic trouble codes. Concurrently, recent advancements in machine learning (ML) have further expanded the capabilities of OBD-II applications, unlocking advanced, intelligent, and data-centric functionalities that significantly surpass those of conventional methodologies. This paper presents a comprehensive investigation into ML-based applications that leverage OBD-II sensor data, aiming to enhance sustainability, operational efficiency, safety, and security in modern vehicular systems. To this end, a diverse set of ML approaches is examined, encompassing supervised, unsupervised, reinforcement learning (RL), deep learning (DL), and hybrid models intended to support advanced driving analytics tasks such as fuel optimization, emission control, driver behavior analysis, anomaly detection, cybersecurity, road perception, and driving support. Furthermore, this paper synthesizes recent research contributions and practical implementations, identifies prevailing challenges, and outlines prospective research directions.

## 1. Introduction

The rapid evolution of intelligent transportation systems (ITS) is characterized by the integration of sensing technologies, data analytics, connectivity, and automation, which together are reshaping the automotive landscape toward safer, more efficient, and environmentally sustainable mobility solutions [1]. Within this framework, the role of in-vehicle diagnostics and data-driven decision-making has become increasingly prominent. First introduced in the mid-1990s, On-Board Diagnostics II (OBD-II) systems [2] represent a foundational element of this transformation by providing standardized access to real-time vehicle performance data through embedded sensors. By facilitating the monitoring of critical operational parameters such as engine metrics (e.g., revolutions per minute (RPM), throttle position, coolant temperature), vehicle dynamics (e.g., speed, acceleration, steering angle), and diagnostic trouble codes (DTCs), OBD-II systems support various smart transportation objectives such as vehicle health management, regulatory compliance, and driving efficiency. While OBD-II was initially designed to monitor vehicle emissions and reduce environmental impact, its use has expanded to encompass a wide range of ITS applications, including eco-driving, predictive maintenance, and driver behavior profiling [3,4].

In parallel, machine learning (ML) has emerged as a key technology for online diagnostics in automotive systems [5], enabling real-time fault detection, failure prediction, and vehicle health monitoring. An online learning framework for real-time sensor fault diagnosis in autonomous vehicles was presented in [6], combining data stream classification with feature ranking to address non-stationary fault behavior. Moreover, the integration of various ML algorithms for vehicle fault detection was proposed in [7]. Furthermore, the shortcomings of traditional battery diagnostics methods under uncertain conditions were discussed in [8], where deep learning (DL) was proposed as a powerful alternative for handling complex and noisy data. The work in [9] proposed an ML-based object classification mechanism for autonomous vehicles to accurately detect and identify various road entities, whereas in [10] the authors introduced an explainable artificial intelligence (XAI) framework to enhance interpretability and efficiency in autonomous vehicle object detection. Additionally, the integration of XAI and multi-sensor fusion in autonomous vehicles was explored in [11]. While much of the existing research has focused on automotive and in-vehicle networks, recent studies have also extended these concepts to a broader range of mechanical systems. For instance, the work in [12] proposed a DL-based method for onboard graded diagnosis of rail corrugation using axle box acceleration signals preprocessed using the empirical wavelet transform (EWT) [13].

In earlier studies, ML has been leveraged to harness the vast amount of data provided by OBD-II systems [14,15]. The use of ML models enables predictive maintenance via early detection of potential faults [16,17], optimization of fuel consumption through real-time eco-driving feedback [18], and accurate emission estimates for pollutants such as NO_*x*_ (nitrogen oxides) and CO_*x*_ (carbon oxides, including CO and CO_2_) [19]. Furthermore, ML-driven OBD-II-based applications play a critical role in profiling driver behavior [20], detecting risky driving patterns, and supporting Advanced Driver-Assistance Systems (ADAS) through enhanced road surface classification and vehicle navigation, particularly in Global Positioning System (GPS)-denied environments such as urban canyons or tunnels. The rise of connected vehicles has been enabled by wireless technologies such as Bluetooth, Wireless Fidelity (Wi-Fi), and ITS-G5 [21], further expanding the role of OBD-II systems and allowing seamless data transmission to cloud platforms for advanced analytics and fleet management. However, this enhanced connectivity introduces cybersecurity risks [22]; OBD-II interfaces may serve as potential entry points for cyberattacks, requiring robust intrusion detection systems (IDS) to safeguard in-vehicle networks.

Despite the growing number of studies applying ML to OBD-II data, there is a lack of unified and systematic reviews that bridge technological advancements with practical automotive applications and emerging industrial needs. Existing surveys are often narrowly focused on specific applications, overlooking cross-domain synergies and deployment challenges. To address this gap, this study aims to provide a comprehensive and application-oriented review of ML-driven OBD-II systems, synthesizing findings across key domains to identify current capabilities, research trends, and future directions relevant to both academia and industry.

### 1.1. Contributions

Building upon the aforementioned observations, this paper aims to deliver a comprehensive examination of recent ML applications within OBD-II systems while evaluating their potential across various automotive domains. The main contributions are as follows:

- **Overview of OBD-II and ML**: A foundational overview of OBD-II system architecture and operational principles is provided, followed by an introduction to ML paradigms suitable for vehicular applications. Emphasis is placed on the synergy between these technologies in enabling intelligent automotive systems.
- **Systematic Exploration of Key Application Domains**: Findings from diverse case studies are discussed to illustrate practical applications of ML-driven OBD-II systems. In this context, a detailed review across critical domains where ML offers substantial advancements is conducted, including the following thematic axes: (i) fuel consumption and energy optimization; (ii) emission control and environmental impact; (iii) driver behavior profiling with personalized feedback; (iv) vehicle health monitoring and anomaly detection; (v) cybersecurity for in-vehicle networks; and (vi) intelligent road perception and driving support. These domains were identified through a systematic literature review across major academic databases such as ACM Digital Library, IEEE Xplore, PubMed, Web of Science, and others. Specifically, we focused on keywords related to ML and OBD-II technology, identifying thematic areas where ML applications are most impactful. Moreover, we selected articles written in English, primarily from peer-reviewed journals, published between 2021 and May 2025, and that directly explored ML-based applications in these areas. Studies that did not center on ML methods or that addressed unrelated OBD-II aspects were excluded from our review.
- **Evaluation of ML Models for OBD-II Data**: The performance of supervised, unsupervised, reinforcement learning (RL), deep learning (DL), and hybrid models is investigated to analyze their suitability, performance tradeoffs, and deployment feasibility in OBD-II environments. In this direction, the benefits and challenges of real-time OBD-II data processing are analyzed, emphasizing the use of low-latency ML algorithms for tasks such as predictive maintenance, eco-driving feedback, and anomaly detection.
- **Lessons Learned, Challenges, Gaps, and Research Directions**: The main lessons derived from the practical deployment of ML models in OBD-II systems are summarized. In addition, critical limitations are identified, including challenges and gaps in model generalization, scalability, computational efficiency, data privacy, and regulatory compliance, and prospective directions for future research are outlined.

**Figure 1:**
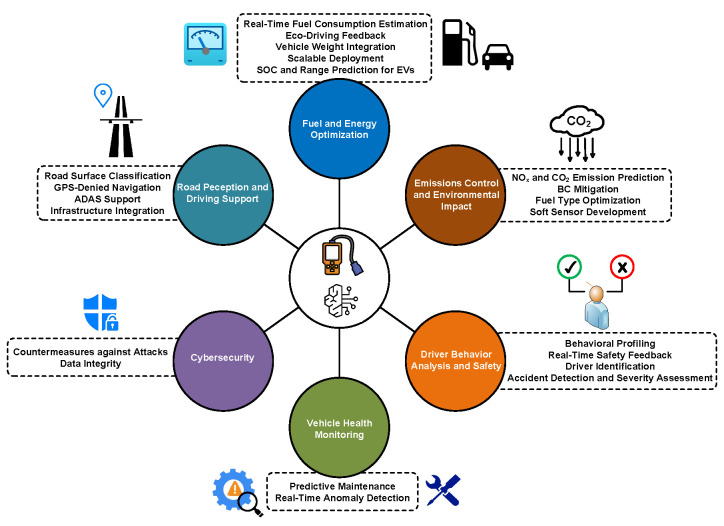
Benefits of machine learning (ML) adoption in On-Board Diagnostics II (OBD-II) systems.

Figure 1 illustrates the six core domains where ML enhances OBD-II functionality, highlighting the transformative potential of data-driven approaches in modern vehicles. It can be seen that ML not only optimizes operational efficiency (e.g., fuel consumption) and environmental sustainability (e.g., emission control) but also strengthens safety and security through driver behavior analysis and cybersecurity.

### 1.2. Previous Review Papers

The recent literature includes several review studies that explore ML applications in the automotive domain. In [20], the authors reviewed the literature on driving behavior analysis, generative AI, predictive maintenance, and driver profiling. Their study underlined the utility of ML and DL techniques in categorizing driver behavior, identifying unsafe driving patterns, and optimizing maintenance scheduling. They also examined the integration of generative AI to deliver real-time personalized driver feedback. Nevertheless, the focus centered on behavior personalization and maintenance, lacking broader discussions on environmental metrics or energy efficiency. The work in [17] surveyed AI-based predictive maintenance and anomaly detection techniques, highlighting XAI, cost reduction, maintenance forecasting, and vehicle health monitoring. While comprehensive in the context of diagnostics and prognostics, the study did not address driver behavior analysis, emission estimation, or fuel optimization through OBD-II interfaces. In [19], the authors tackled NO_*x*_ emission source identification in heavy-duty diesel vehicles (HDDVs) using OBD-II data and ML methods. Their framework incorporated data processing and blended analytics for emission source recognition. Despite its strong domain specificity and policy relevance, their study was limited to emission analysis for a small vehicle subset and did not encompass other key OBD-II applications such as driving behavior or ITS integration. The review in [23] addressed ML-based traffic–air quality (TAQ) models, with an emphasis on high-resolution environmental modeling in European regions. In particular, the study analyzed data such as vehicle sensor data from both real and simulated sources in order to capture the spatiotemporal variability of urban traffic emissions. Although insightful for urban pollution modeling, the study diverged from direct vehicle-level OBD-II analysis and did not address ML-driven vehicular control or driver profiling. In [18], a detailed review was presented on eco-safe driving behavior modeling. The authors examined ML-based driving profiling characterization using OBD-II data augmented with GPS, environmental, physiological, and contextual features. Furthermore, their study compared shallow and deep learning methods and investigated fuel consumption prediction using regression and sequence models such as Long Short-Term Memory (LSTM) networks. However, the review was more focused on simulations than on end-to-end ML deployments deploying real-world OBD-II data in ITS optimization scenarios. Lastly, the work in [24] provided a broad overview of ML algorithms for driver behavior identification, including fuzzy logic, genetic algorithms, clustering, and RL. The emphasis was placed on algorithmic diversity and future directions in personalized modeling. However, the study did not sufficiently discuss OBD-II applications in environmental sustainability or intelligent transport frameworks. In summary, the aforementioned works, which are summarized in Table 1, have a limited scope, targeting specific aspects of the automotive data ecosystem such as driver behavior modeling, predictive maintenance, or emissions analysis. In contrast, the present paper contributes a holistic review of ML and DL applications leveraging OBD-II data.

**Table 1:** Relevant review and survey papers.

Reference	OBD-II-Centric	Energy Consumption	Emission Control & Environmental Impact	Automotive Behavior & Driver Analysis	Anomaly Detection & Cybersecurity	Road Perception & Driving Support
Shirole et al., 2025 [20]	✗	√	✗	√	✗	✗
Mahale et al., 2025 [17]	✗	✗	✗	✗	√	✗
Cao et al., 2025 [19]	√	√	√	✗	✗	✗
Du et al., 2025 [23]	✗	✗	√	✗	✗	✗
Jain et al., 2023 [18]	√	√	✗	√	✗	✗
Malik et al., 2023 [24]	✗	✗	✗	√	✗	✗
This paper	√	√	√	√	√	√

### 1.3. Structure

The remainder of this paper is organized as follows: Section 2 introduces OBD-II technology and its integration with ML; Section 3 explores methods for monitoring and optimizing energy consumption; Section 4 examines approaches for emission control and environmental impact assessment; Section 5 delves into safety issues, behavioral modeling, and driver analysis; Section 6 addresses anomaly detection and cybersecurity challenges; Section 7 highlights the application of ML in intelligent road perception and driving assistance; Section 8 summarizes lessons learned and synthesizes key insights; Section 9 discusses implementation challenges, identifies research gaps, and suggests promising directions for future work; finally, Section 10 provides concluding remarks.

## 2. Overview of OBD Technology and ML

### 2.1. OBD-II System and Regulatory Framework

OBD refers to a standardized framework that enables external tools to access operational data from vehicle systems, primarily the engine and powertrain. Initially introduced in the 1980s for emission monitoring, early OBD implementations were proprietary and limited. Standardization in the mid-1990s significantly enhanced diagnostic and emission-monitoring capabilities [25] while extending support to motorcycles and two-wheelers. In the European Union (EU), a series of regulations define the structure, access, and obligations of OBD systems. EC Regulation No. 715/2007 under the Euro 5 and Euro 6 emission standards set the basis by mandating fault code reporting and ensuring data access for independent repairers [26]. Regulation 2018/858 later introduced a harmonized EU type-approval system and broadened third-party access to OBD-II data [27]. The upcoming Euro 7 regulation aims to enhance remote diagnostics and telemetry, supporting integration into connected-vehicle platforms [28]. Separately, EU Regulation 2019/631 [29] sets CO_2_ emission targets for new passenger cars and light commercial vehicles, mandating a 15% reduction by 2025 and reductions of 37.5% (cars)/31% (light commercial vehicles) by 2030 relative to 2021 levels.

At the technical level, OBD-II [30] implements the application-layer protocol defined in SAE J1979 [31], specifying diagnostic modes, message structure, and semantics. It typically operates over the Controller Area Network (CAN) as per ISO 15765-4 [32], functioning as a request–response system in which external scanners query data from Electronic Control Units (ECUs) either directly or via a central gateway. Key modes include: Mode 01 (real-time powertrain data), Mode 03 (emission-related DTCs), Mode 04 (DTC clearing and malfunction indicator lamp (MIL) reset), and Mode 09 (vehicle identification). Parameters are identified via unique Parameter IDs (PIDs) and span the following:

- Engine/powertrain: RPM (PID 0C), throttle (PID 11), coolant and intake air temperature (IAT) temperatures, manifold pressure.
- Fuel system: short/long fuel trims, fuel level, equivalence ratio.
- Emissions: O_2_ sensor outputs, catalyst temperatures.
- Vehicle dynamics: speed (PID 0D), wheel speeds, steering angle.
- Maintenance: DTCs, time since engine start.

Sampling rates vary with factors such as the number of PIDs requested, protocol limitations, and ECU load. Typical sampling rates range from 1 Hz up to 100 Hz, while direct access to raw CAN frames is able to reach rates near 1 kHz. Although CAN is the dominant communication protocol used in OBD-II, other protocols may be deployed depending on the application [32,33]. For instance, FlexRay supports high-reliability deterministic communication for safety-critical applications such as ADAS, while automotive Ethernet is increasingly used for bandwidth-intensive services such as infotainment. CAN messages are prioritized using identifier-based arbitration, meaning that real-time control data can take precedence over diagnostic traffic. If present, gateways can mediate access, translate between protocols, or enforce authentication policies, and are often implemented using Unified Diagnostic Services (UDS) as defined in ISO 14229 [34].

**Figure 2:**
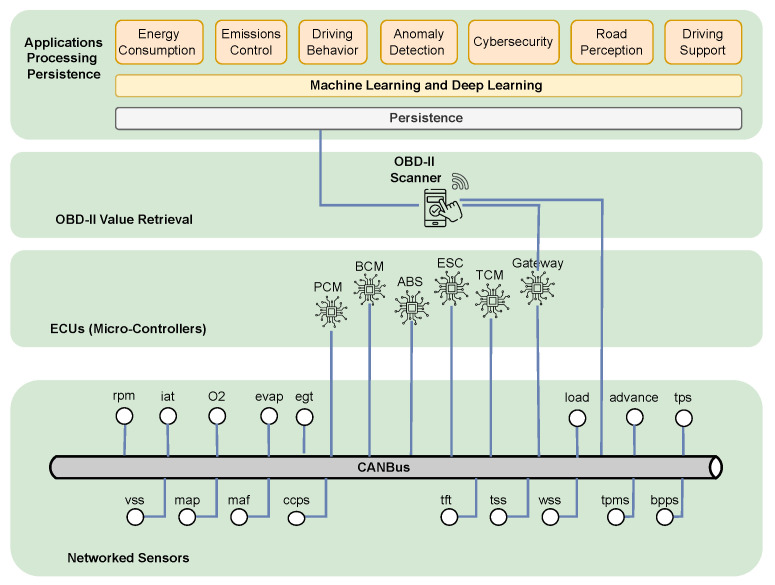
System architecture of an OBD-II diagnostic interface.

**Figure 3:**
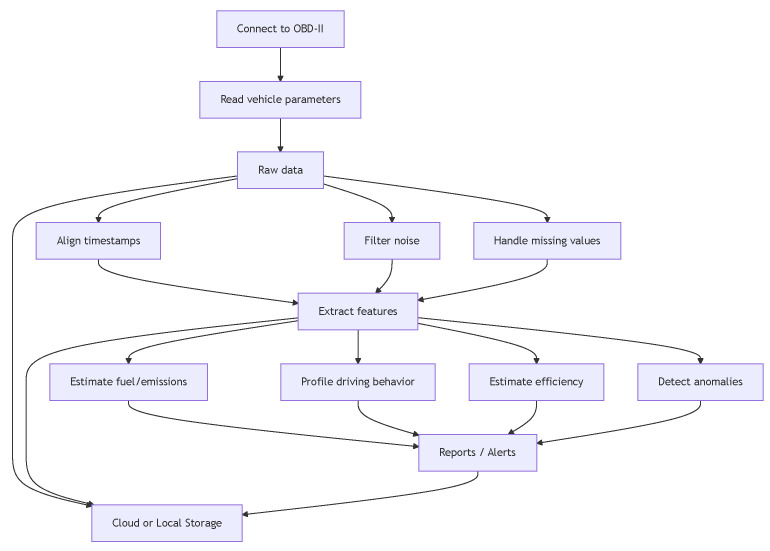
Modular flow for OBD-II data retrieval and processing.

Physically, the OBD-II interface connects via a standardized 16-pin connector, typically located under the vehicle’s dashboard. Diagnostic tools interface with external applications either via wired link or using wireless protocols such as Bluetooth. As depicted in Figure 2, the bus (typically a CANBus) allows for communication between the data producers (sensors) and consumers (ECUs and OBD-II diagnostics). Sensors include RPM, IAT, O2 (lambda), evap (evap system pressure), exhaust gas temperature (EGT), calculated load, advance (ignition timing advance), throttle position sensor (TPS), vehicle speed sensor (VSS), manifold absolute pressure (MAP), mass airflow (MAF), crankshaft and camshaft position sensors (CCPS), transmission fluid temperature (TFT), transmission input/output shaft speed (TSS), wheel speed sensor (WSS), tire pressure monitoring (TPMS), and brake pedal position (BPSS) sensors. These sensors are available either through standard OBD-II or through manufacturer-specific extended PIDs over CANBus. The data are requested or retrieved by vehicle ECUs, including the Powertrain Control Module (PCM) for engine performance and emission management, the Transmission Control Module (TCM) for clutch control, the Anti-lock Braking System (ABS), the Electronic Stability Control (ESC) related to wheel speed, yaw, and lateral motion, and the Body Control Module (BCM). Parameter values can be retrieved by OBD-II either directly or through a gateway. As shown in Figure 3, OBD-II data processing follows a modular pipeline: connection to the vehicle, raw data collection, preprocessing (e.g., alignment, noise filtering, imputation), and feature engineering. The resulting features feed inference modules for tasks such as fuel/emission estimation, driving behavior analysis, efficiency assessment, and anomaly detection. Raw data and/or extracted features can persist either locally (e.g., on a connected mobile device) or in cloud infrastructure, allowing for post hoc analysis and model retraining. While mandatory for emission monitoring in internal combustion engine vehicles, OBD-II is not universally required in electric vehicles (EVs). Most EVs include OBD-II ports but lack traditional engine PIDs (e.g., RPM, fuel rate), instead using proprietary or extended protocols to report battery-related metrics such as battery state of charge (SoC), voltage, temperature, and regenerative braking activity. To address diagnostics for EVs, Zero Emission Vehicles (ZEVs), and Plug-in Hybrid Electric Vehicles (PHEVs), the SAE J1979-3 standard was introduced [35].

### 2.2. ML for OBD-II-Based Vehicle Intelligence

Conventional OBD-II applications rely on rule- or threshold-based approaches in which predefined DTCs or static thresholds for sensor parameters trigger alerts for specific issues (e.g., emission violations or mechanical faults). However, these methods are limited to detecting known issues based on predetermined criteria, and may struggle with the complexity of real-world driving conditions that involve multiple interacting variables (e.g., engine load, vehicle speed, throttle position). Moreover, OBD-II operations are primarily reactive, identifying faults only after they occur (e.g., triggering a DTC when a sensor reading exceeds a threshold). This approach cannot predict potential issues, leading to delayed maintenance and increased repair costs. In addition, conventional OBD-II systems offer limited real-time feedback, usually confined to basic alerts (e.g., check engine light) or static eco-driving suggestions based on straightforward metrics such as fuel consumption rate. Consequently, they are unable to dynamically adapt and optimize performance under changing driving conditions.

With growing demand for OBD-II applications that can operate effectively under dynamic conditions (e.g., varying vehicle loads, driver behavior, and traffic), ML algorithms are significantly improving the ability to process and analyze the complex and noisy sensor data provided by OBD-II systems. ML supports both post hoc analysis and real-time inference, often requiring low-latency processing to be performed at the edge or within the vehicle. By analyzing large volumes of OBD-II data, ML can reveal complex nonlinear relationships related to engine performance, system faults, and external factors, enabling predictive maintenance to forecast faults before they occur [17,20]. ML also delivers dynamic personalized feedback for applications such as fuel optimization [18,24] and eco-driving [19,23]. Additionally, DL models can analyze sensor inputs such as throttle position and steering angle to accurately profile driver behavior and detect risky patterns [18,24]. However, the bidirectional connectivity of OBD-II interfaces through Bluetooth or telematics introduces cybersecurity vulnerabilities, making ML-based intrusion detection systems an important research focus [36]. The success of ML approaches depends heavily on high-quality, structured, and temporally aligned datasets, which can be compromised by sensor noise, latency, or degradation. Therefore, thorough preprocessing via techniques such as denoising, imputation, normalization, and temporal alignment is critical to improve the signal-to-noise ratio (SNR) and enable effective learning.

The application of ML in OBD-II systems can be categorized into several methodological paradigms, each offering distinct capabilities and advantages [20,21,22,23,24,36]. Supervised learning models rely on labeled datasets to map inputs such as sensor readings to outputs such as fault types or emission levels. These models are commonly used for classification and regression, are well-suited for structured diagnostic tasks, and offer high interpretability, which is crucial in automotive contexts. Popular algorithms include Decision Tree (DT), Support Vector Machine (SVM), Random Forest (RF), k-Nearest Neighbors (kNN), and Gradient Boosting Machine (GBM). By contrast, unsupervised learning models operate without labeled data and use clustering techniques such as k-means or hierarchical clustering to reveal hidden patterns, detect anomalies, or profile driver behavior, which is useful for early fault detection and operational monitoring. Moreover, RL models train agents to interact with their environment by optimizing long-term rewards; although less common in diagnostic tasks, RL shows promise for optimizing adaptive vehicular subsystems that must respond dynamically to varying internal and external conditions. Furthermore, DL models such as Recurrent Neural Networks (RNNs), LSTM networks, and Convolutional Neural Networks (CNNs) excel at learning from high-dimensional and temporally correlated sensor data, allowing them to capture complex dynamics and spatial dependencies. However, they typically require large datasets and substantial computational resources, and often lack interpretability. Additionally, hybrid models integrating multiple ML paradigms or combine ML with optimization techniques (e.g., genetic algorithms) can enhance accuracy, robustness, and efficiency, particularly for complex multi-objective tasks such as coordinated subsystem diagnostics or performance optimization. Lastly, probabilistic and rule-based models offer mechanisms for modeling uncertainty and ensuring regulatory compliance. Probabilistic approaches enable robust inference under partial observability by capturing dependencies between variables, while rule-based systems rely on interpretable human-defined logic, making them suitable for applications where transparency and compliance with regulatory standards are essential.

**Figure 4:**
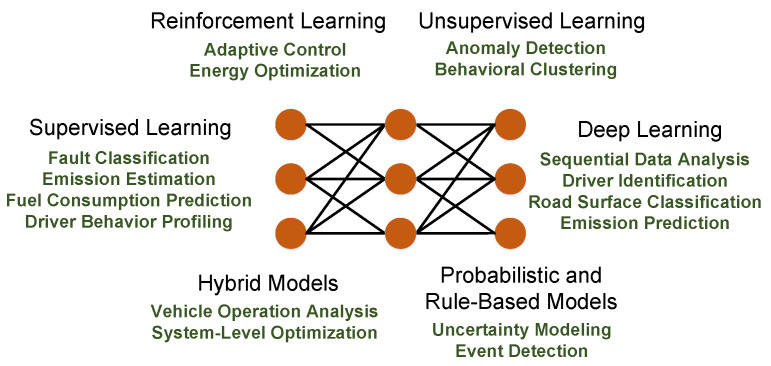
ML categories and indicative OBD-II-based applications.

Figure 4 categorizes ML paradigms and maps them to OBD-II applications, providing a clear framework for understanding their roles in vehicular intelligence. The choice of an appropriate model depends on the specific objectives, data availability, and computational constraints. For instance, supervised learning (e.g., RF) dominates fuel consumption prediction due to its interpretability, while DL (e.g., LSTM [5]) excels in modeling temporal patterns for emission monitoring. This figure also highlights the emerging potential of RL for adaptive control (e.g., eco-driving optimization) and the need for hybrid models to tackle complex multi-objective tasks such as system-level diagnostics.

## 3. Monitoring, Estimation, and Optimization of Energy Consumption

Energy consumption is a critical aspect of vehicle management, impacting operational costs and environmental sustainability. Thanks to the synergy between OBD-II and ML, real-time monitoring, estimation, and optimization of energy usage are becoming increasingly viable. This section examines recent ML-based approaches, which are summarized in Table 2.

**Table 2:** Overview of ML-based approaches for fuel and energy consumption optimization.

Reference	Objective	ML Models	Driving Environment	Type of Vehicle and Fuel	OBD or OBD-Derived Parameters	Performance Metrics
Yen et al., 2021 [15]	Prediction of fuel consumption and identification of abnormal driving behaviors	FFB, RNN, and Elman Neural Networks	Regular and steep mountain roads, varying road conditions	Not explicitly mentioned	Engine RPM, vehicle speed, engine load, throttle position, coolant temp, air flow rate	RMSE and correlation coefficient (γ)
Abediasl et al., 2024 [37]	Real-time estimation of fuel consumption	RF and ANNs	Urban and highway driving conditions; includes intersections, stop signs, pedestrian crossings	HEV, PHEV, turbocharged gasoline, gasoline V8	Engine load, engine speed, intake MAP, throttle position, air-fuel equivalence ratio, engine coolant temperature	RMSE and NMAE
Fan et al., 2024 [38]	Reducing fuel consumption, promoting eco-driving, and providing driver feedback and alerts	LSTM-Conv and ANNs	Regular and mountain roads, traffic, varied terrains	Diesel-powered HDTs	Engine speed, vehicle speed, throttle position, fuel factor, and engine load	MAE, MAPE, RMSE, and R^2^ score
Hu et al., 2024 [39]	Monitoring vehicle conditions, ensuring safety, and optimizing fuel efficiency	LSTM neural network	Regular operational conditions	Diesel-powered HDTs	Vehicle speed, engine speed, air intake volume, fuel flow, and accelerator pedal depth	MAPE, MAE, MSE, and R^2^ score
Kabir et al., 2023 [40]	Monitoring and estimation of fuel consumption in urban traffic	LSTM neural networks	Signalized intersections with traffic states (queued, free-flowing, acceleration, deceleration)	Not explicitly mentioned	Speed and FCR	RMSE, MAE, and SMAPE
Rykala et al., 2023 [41]	Cost-effective, real-time monitoring of fuel consumption using low-cost technology	Multivariate regression model, DT (CART algorithm), and ANNs	Varied terrain and speed limits	Gasoline-powered vehicle	Engine speed, vehicle speed, and engine load	MSE, MAE, and MRAE
Eissa et al., 2023 [42]	Predicting the SOC and the RDR	Nonlinear SVR model with a RBF kernel	Rural driving conditions	EV	Battery current, battery voltage, battery temperature, ambient temperature, EV speed, SOC	MAE and R^2^ score

The work in [15] developed a universal OBD-II module integrated with DL to enable eco-driving analysis, targeting improved efficiency and reduced fuel consumption across various vehicle models. The module supported multiple CAN protocols (ISO-15031 [43], ISO-27145 [44], SAE-J1939 [45]) and was tested on a Mazda 3 (1999cc), Mitsubishi Lancer (1798cc), and Toyota Vios (1496cc) under diverse road conditions in Taiwan, including urban and mountainous terrains. Real-time data were transmitted via Bluetooth from the module to a smartphone, then to a computer for processing. Recorded parameters included engine displacement, air flow, coolant temperature, engine load, ignition timing, RPM, speed, throttle position, and control module voltage. For instantaneous fuel consumption prediction, three DL models were used: Feed-Forward Backpropagation (FFB), RNN, and Elman Neural Network, with the Elman model achieving the lowest RMSE of 3.672 L/km and the highest correlation coefficient (γ = 98.27%). Mountainous driving notably increased consumption, with the Toyota Vios consuming up to 45.37 L/100 km. Additionally, an intuitive GUI provided real-time feedback on eco-driving, flagging inefficient behaviors such as excessive acceleration and idling. DL models significantly outperformed traditional regression methods, achieving over 96% accuracy.

In [37], an RF- and Artificial Neural Network (ANN)-based approach was presented for real-time fuel consumption estimation. The analysis leveraged OBD-II parameters (i.e., engine load, engine speed, MAP, throttle position, air–fuel equivalence ratio, and coolant temperature) collected via the CAN data logger (CANedge2, CSS Electronics, Aabyhoej, Denmark), whereas fuel consumption was measured using a Sentronics FlowSonic ultrasonic fuel flow meter (Sentronics Ltd., Bournemouth, United Kingdom). Moreover, the experimental setup involved four fleet vehicles: a hybrid electric vehicle (HEV) (Ford Fusion Hybrid, 2.5 L), a PHEV (i.e., Ford Escape sport utility vehicle (SUV), 2.5 L), a turbocharged gasoline SUV (Ford Escape S, 1.5 L), and a gasoline pickup truck (Ford F-350, 6.2 L) driven under two real-world conditions: a 100 km highway route and a 20.5 km urban route in Edmonton, Canada. The results showed that RF outperformed ANN for instantaneous fuel consumption estimation in both scenarios, with highest accuracy in highway driving. For the Ford Fusion Hybrid, RF achieved a Root Mean Square Error (RMSE) of 4.68 g/min and a Normalized Mean Absolute Error (NMAE) of 3.9%, while the ANN reached 9.71 g/min and 11.9%. Similarly, for the Ford F-350, RF yielded 11.01 g/min (NMAE: 1.8%), outperforming the ANN’s 21.21 g/min (NMAE: 9.3%). Urban driving produced higher errors due to stop-and-go behavior, yet RF still performed well, achieving 4.17 g/min (NMAE: 2.6%) for the Ford Escape PHEV and 6.71 g/min (NMAE: 5.9%) for the Ford Escape S. ML models also outperformed ECU-based estimation, with ECU estimates showing higher errors (NMAE: 16.4% for the Ford Fusion Hybrid; 32.6% for the Ford Escape S).

The work in [38] addressed fuel consumption estimation in heavy-duty trucks (HDTs) by incorporating vehicle weight as a key factor. Five models were evaluated: Vehicle-Specific Power (VSP), VT-Micro, an Engine Output Power (EOP)-based model, an ANN, and a hybrid LSTM–Convolutional network (LSTM-Conv). Using over 4 million second-by-second OBD records from 162 HDTs (81,761 km, 1180 h) in Shandong Province, key parameters included engine and vehicle speed, throttle position, fuel factor, and engine load. Real-time weight data were collected at loading/unloading sites and integrated via cloud transmission. The results showed a nonlinear relationship between weight and fuel consumption: 15–25 ton trucks consumed 290% more fuel than 5-ton trucks, while 45–55 ton trucks showed a 755% increase under low-speed positive acceleration. Weight impact was strongest at low speeds but diminished at high speeds due to drag. DL models outperformed traditional ones, with LSTM-Conv achieving the lowest Mean Absolute Percentage Error (MAPE) (9.81% for fuel consumption rate (FCR), 1.49% for trip economy), far better than VSP’s 182.45%. ANN models also improved accuracy. Models using weight data showed greater stability across conditions, while traditional models under- or overestimated consumption depending on load. The LSTM-Conv model proved highly accurate and robust, making it ideal for fleet fuel management.

The work in [39] introduced a novel truck mass estimation method using OBD data and an LSTM neural network, aiming to improve convenience, scalability, and accuracy over existing techniques. Designed to support road safety, overload monitoring, and fuel efficiency, the study addressed risks such as extended braking distance and increased accident rates. In collaboration with transport companies in Yulin, China, data were collected from three HDTs over 175 days (224 trips), with ground truth mass obtained via weighing scales. Seven OBD-II parameterswere used as LSTM model inputs: vehicle speed, engine output torque, fuel flow, engine speed, pedal depth, friction torque, and air intake. The estimation ran locally on the vehicle using Python and TensorFlow for real-time processing. Performance was evaluated using the MAPE, Mean Squared Error (MSE), MAE, and Coefficient of Determination (R^2^). The LSTM achieved an average error of 2.3%, outperforming traditional methods such as Extended Kalman Filtering (EKF) and Electronic Parking Brake (EPB)-based weight estimation. Local deployment provided faster response and greater reliability than cloud-based systems. The study combined OBD data with external signals (trip data, road conditions, weigh station readings) to enhance accuracy. A proprietary dataset with high-frequency OBD sampling and 355 validated trip segments ensured real-world relevance. Model evaluation across diverse traffic and road conditions confirmed the LSTM model’s superior accuracy and generalization.

In [40], the impact of traffic congestion and delays on fuel consumption at signalized intersections was examined using OBD and Probe Vehicle Trajectory (PVT) data. LSTM neural networks designed for time series data were used to predict FCRs based on key OBD parameters such as speed, elevation, and fuel use. An Autoregressive Integrated Moving Average (ARIMA) model served as the baseline. Developed in Python with TensorFlow and scikit-learn, the system included Density-Based Spatial Clustering of Applications with Noise (DBSCAN)-based outlier detection and feature normalization. Model performance was evaluated using the RMSE, MAE, and Symmetric MAPE (SMAPE). The LSTM model significantly outperformed ARIMA, achieving a 31.7% reduction in RMSE, 45.5% in MAE, and 49.8% in SMAPE, demonstrating its superior accuracy in predicting fuel consumption. The LSTM model was deployed locally in the vehicle for real-time processing instead of relying on cloud-based systems. The dataset combined private and crowdsourced data from two regions: OBD data from a compact vehicle in Detroit, Michigan (collected over 6 h, with 12,885 observations), and PVT data from Chattanooga, Tennessee (collected from GPS trajectories at ten signalized intersections on Shallowford Road). The study analyzed the relationship between vehicle delay and fuel consumption, observing 352 vehicles at intersections. The results showed that congestion and inefficient signal timing significantly raised fuel use, especially during peak hours.

The work in [41] developed a cost-effective method for real-time vehicle fuel consumption monitoring using a low-cost OBD-II interface. Three predictive models were assessed: multivariate regression, DTs (CART), and ANNs. A real-world test was conducted on a 2016 Mazda 3 (SkyActiv-G 2.0) over a 320 km route from Warsaw to Rzeszów, Poland, covering diverse road conditions such as urban streets and highways as well as varying terrain elevations. Data were collected through a Vgate iCar Pro Wi-Fi OBD-II adapter (Vgate Technology Co., Ltd., Shenzhen, China) and the Torque Pro application, capturing parameters such as engine speed (RPM), vehicle speed, load, fuel consumption, gear position, and acceleration. GPS-based slope data from altitude changes helped to analyze the impact of road incline. Among the models, multivariate regression delivered the best results (MSE = 3.19, MAE = 1.25, Mean Relative Absolute Error (MRAE) = 0.13), outperforming DTs (163% higher MSE, 21% higher MAE, 92% higher MRAE) and ANNs (73% higher MSE, 10% higher Mean Absolute Error (MAE)). Sensitivity analysis showed that vehicle speed, engine load, and RPM were the most impactful for ANNs, while the regression model identified acceleration as the dominant factor, estimating a 0.94 L/100 km increase in fuel consumption per 1 m/s^2^ rise in acceleration.

In [42], an ML-based framework for accurately predicting the SoC and remaining driving range (RDR) of EVs in rural environments was introduced, tackling challenges such as limited charging infrastructure and cold weather effects. The primary objective of this work was to enhance SOC prediction accuracy, allowing rural EV drivers to plan their trips more efficiently and mitigate range anxiety in regions where public charging stations are scarce. Three factors influencing EV range were considered: vehicle parameters, driver behavior, and environment. In this context, real-world driving cycle (RDC) data were collected over a two-week period from 20 drivers in the Upper Cumberland region, Tennessee, USA using two Nissan Leaf models: a 2019 Nissan Leaf SL Plus and a 2020 Nissan Leaf SV Plus. The dataset was recorded using a Hem Data OBD Mini Logger (Altair Engineering, Inc., Troy, MI, USA), which captured essential OBD parameters such as the battery current, battery voltage, battery temperature, ambient temperature, EV speed, and SOC. Moreover, this work leveraged a nonlinear Support Vector Regression (SVR) model with a radial basis function (RBF) kernel to predict SOC while considering the complex dependencies between OBD parameters and battery discharge behavior. The SoC model achieved high accuracy (R^2^ = 0.95, MAE = 2.4%), performing well despite driver and road variations. The RDR was estimated from SoC predictions, with the average error under 2%. Analysis showed slightly better accuracy for models trained on the Nissan Leaf SL Plus data, which was attributed to training bias.

## 4. Emission Control and Environmental Impact

Emission monitoring plays a key role in the design of strategies aimed at reducing air pollution and improving air quality. In parallel, emission prediction enables the implementation of proactive measures to prevent environmental degradation and ensure regulatory compliance. Notably, NO_*x*_ and CO_*x*_ differ significantly in terms of their formation mechanisms, environmental impact, and control strategies. NO_*x*_ is particularly challenging to manage due to its formation under high-temperature combustion and the stringent regulations associated with its adverse health and ecological effects. In contrast, CO is more readily controlled, especially in gasoline engines, while CO_2_ is addressed indirectly through fuel economy and carbon emission targets. OBD-II systems monitor pollutants using onboard sensors, which can identify malfunctions in emission control components such as Selective Catalytic Reduction (SCR) systems for NO_*x*_ and three-way catalytic converters for CO. Tighter regulatory scrutiny makes NO_*x*_ monitoring of particular importance, especially for diesel vehicles. Despite their widespread deployment, traditional OBD-II systems rely on direct sensor data and threshold-based diagnostics, which are constrained by sensor reliability and their limited capacity to capture the dynamics among driving behavior, engine load, and environmental conditions. To address these limitations, recent studies have applied ML techniques to monitor and predict vehicular emission. A detailed overview of these ML-based approaches can be found in Table 3, Table 4 and Table 5.

### 4.1. Identification, Monitoring and Prediction of NO_x_ Emissions

HDDVs are a major NO_*x*_ source in road transport due to their high fuel use and extended driving ranges. The work in [46] aimed to accurately calculate NO_*x*_ emission factors using an OBD-based online monitoring dataset in conjunction with the COPERT model [47]. To overcome COPERT’s real-world shortcomings, the authors proposed a two-stream DL architecture integrating time series and time–frequency features through a ResNet50 backbone enhanced with convolutional block attention modules (CBAM). A range of signal processing and ML methods were used, including Spearman rank correlation for feature selection, continuous wavelet transformation for time–frequency representation, and deep CNNs for regression modeling. OBD data were collected from a diesel vehicle (WEICHAI WP12.375E51), including parameters such as engine speed, water temperature, downstream NO_*x*_, gas flow rate, urea tank temperature, accelerator position, output torque percentage, oxygen concentration, and ambient temperature. These were augmented with derived variables such as fuel consumption (from portable emission measurement system (PEMS) or EPA reports), fuel and exhaust density, and stoichiometric ratios. The proposed model, termed HI_TTFTS, was evaluated using MAE, MAPE, and RMSE metrics. Experimental results demonstrated significant improvements over baseline models. Specifically, the HI_TTFTS model achieved an MAE of 0.0077 g/km, MAPE of 7.38%, and RMSE of 0.0137 g/km, outperforming alternative approaches such as SVR (MAE: 0.0864), ANN (MAE: 0.0229), and standard ResNet50 (MAE: 0.0156).

**Table 3:** Overview of ML-based approaches for identification, monitoring and prediction of NO_*x*_ emissions.

Reference	Objective	ML Models	Driving Environment	Type of Vehicle	OBD or OBD-Derived Parameters	Performance Metrics
Xu et al., 2024 [46]	Accurate calculation of NO_*x*_ emission factor on the OBD online monitoring dataset using the COPERT model	ResNet, CBAM, SVR, ANN, CNN	Complex conditions influenced by driving behavior and external environment	HDDV (WEICHAI WP12.375E51 engine)	Engine speed, downstream NO_*x*_, atmospheric pressure, exhaust gas flow rate, urea tank temp, gas pedal opening, FCR	MAE, MAPE, RMSE
Ge et al., 2023 [48]	Identification of malfunctioning or tampered SCR systems	RF, LR	Urban, rural, and motorway segments with speed thresholds	18-ton trucks, 4.5-ton pickups, 19-seat bus (diesel engines)	Engine speed, vehicle speed, exhaust flow, NO_*x*_-specific emissions, coolant temperature	Total accuracy, recall, precision, null accuracy
He et al., 2022 [49]	Timely identification and prediction of excessive NO_*x*_ emissions	PPCA, kNN, NMF, RF, SVM, GBDT	Variable driving conditions	Diesel-powered vans, trucks, semi-tractors	Speed, atmospheric pressure, torque, fuel flow, coolant temp, NO_*x*_ sensor outputs	R^2^, MAPE, RMSE, precision, recall, F1-score
Li et al., 2024 [50]	Analysis of conformity of NO_*x*_ and PN emissions, and reliability of OBD-derived data	Statistical techniques	Long distance, heavy-duty logistics delivery, freeway, urban, suburban roads	N3 HDDVs (China-VI compliant)	NO_*x*_ concentrations, speed, engine speed, fuel flow rate, engine power, torque, ambient temp, barometric pressure	Statistical evaluation tests
Liu et al., 2024 [51]	Correction of NO_*x*_ concentration during DPP in NO_*x*_ sensors	RF, MLP, LSTM	Urban roads, rural roads, and motorways	N3 and M3 HDDVs	Speed, barometric pressure, engine torque, friction torque, SCR NO_*x*_ sensor outputs, DPF pressure, reactant allowance, mileage	R^2^, RMSE, MAE
Xu et al., 2021 [52]	Prediction of NO_*x*_ emissions considering road conditions, driving behavior and dynamics	Temporal fusion transformer-GRU, BPNN	Actual road operating conditions	Diesel vehicle	Torque percentage, water temp, fuel temp, oil temp, downstream oxygen percentage, urea tank level, vehicle speed, gas pedal opening, downstream NO_*x*_	MAE, RMSE
Yang et al., 2024 [53]	Prediction of NO_*x*_ and PN emissions via soft sensor monitoring	GA-GRU, LSTM, SVM, BPNN, SGD, GBDT	Urban, suburban and highway areas (20%, 25%, 55%)	Various HDTs	Coolant temp, vehicle speed, MAF, oil temp, fuel rate, catalyst temp, DPF delta/output pressure	R^2^
Zhao et al., 2024 [54]	Surveillance and prediction of NO_*x*_ emissions under various driving conditions	Seq2Seq neural network (Bi-GRU, attention mechanism, ITL)	Highways outside city centers; acceleration, deceleration, cruise, idle states	HDDV	Instantaneous speed, acceleration, cooling water temp, throttle pedal degree	MAPE, RMSE, MAE

The work in [48] focused on emission monitoring, with an emphasis on ensuring compliance with China VI regulations. China’s standards closely mirror Euro 6 but impose more stringent limits on key pollutants such as NO_*x*_ and particulate matter. More precisely, this work focused on identifying vehicles with malfunctioning or tampered SCR systems that result in elevated NO_*x*_ emissions. The RF technique was employed for detecting and classifying high NO_*x*_ emissions, while Logistic Regression (LR) was used as a benchmark. Data monitoring and analysis were based on cloud-enabled wireless transmission, with data sent to centralized monitoring systems in real time via the Remote Emission Management Vehicle Terminal (REMVT). Field experiments were conducted on four HDDVs under both controlled and variable real-world conditions. The tested vehicle types included an 18-ton truck, a 4.5-ton pickup, and a 19-seat bus. GPS signals and environmental variables such as temperature, pressure, and humidity were integrated with OBD data such as engine speed, vehicle speed, exhaust flow, NO_*x*_-specific emissions, and engine coolant temperature. Data processing took place within a Linux environment using a Remote Operating System (ROS). Additionally, the performance of the applied techniques was evaluated using metrics such as total accuracy, recall, precision, and null accuracy. Based on the results, the RF classifier outperformed LR, with its higher accuracy (0.940), precision (0.672), and recall (0.485) demonstrating greater robustness and accuracy.

To identify excessive NO_*x*_ emissions in a timely fashion, the work in [49] employed Probabilistic Principal Component Analysis (PPCA), kNN, Non-Negative Matrix Factorization (NNMF), RF, SVM, and Gradient Boosting Decision Trees (GBDT). These methods were used to identify excessive emission, enhance the quality of OBD data, and predict NO_*x*_ levels. The processing environment consisted of an OBD monitoring system that integrated hardware components such as sensors and ECUs with software tools such as diagnostic codes and algorithms for data prediction and imputation. In this work, NO_*x*_ data were combined with variables such as vehicle speed, atmospheric pressure, engine output torque, and engine speed. Specific OBD parameters such as speed, atmospheric pressure, net output torque, engine fuel flow, coolant temperature, and SCR NO_*x*_ sensor readings were recorded continuously over time intervals of approximately 30 s. Experiments were carried out under variable driving conditions using four HDDVs, including vans, trucks, and heavy semi-tractors. Evaluation using the MAPE, RMSE, and R^2^ metrics indicated that PPCA provided more accurate predictions than NNMF, although NNMF appeared to be the more stable predictor overall.

The real-world gaseous and particulate emission characteristics of HDTs complying with the China VI standard were investigated in [50]. The objectives of this study were to analyze the on-road conformity of vehicle NO_*x*_ and particle number emissions, calculate and compare emission factors in relation to vehicle features and operating conditions, and assess the reliability and accuracy of NO_*x*_-related data derived from OBD systems. The OBD data were compared against PEMS data, while the statistical signal processing methods included the Mann–Whitney U test, Kruskal–Wallis one-way analysis of variance, Spearman’s rank correlation test, and Pearson correlation coefficient. These techniques were applied both locally in the vehicle and in a cloud-based context. Experiments were conducted in Shenzhen, China, and a total of 21 N3-class HDDVs were tested. The driving conditions during the tests were characterized by long-distance heavy-duty logistics operations. Conditions varied significantly in terms of vehicle features, operational modes, compliance status of the vehicles (qualified or unqualified), and road types, including freeways, urban roads, and suburban roads. Based on the results, a notable 38.1% of the vehicles failed to meet regulatory emission standards, while approximately 43% provided unreliable OBD-based NO_*x*_ data.

The study in [51] focused on correcting NO_*x*_ concentration measurements during the dew point protection (DPP) phase of onboard NO_*x*_ sensors. Spearman correlation analysis, RF, Multi-Layer Perceptron (MLP), and LSTM networks were used. These techniques served to assess monotonic relationships between variables and to predict NO_*x*_ concentrations. The applied methods were deployed both locally in the vehicle and in a cloud-based environment. Among the considered OBD parameters were the vehicle speed, friction torque, fuel flow rate, air flow rate, SCR upstream NO_*x*_ sensor output, SCR inlet temperature, diesel particulate filter (DPF) differential pressure, reactant allowance, and fuel level. These were integrated with data from PEMS, exhaust flow signals, weather conditions, and GPS-based position signals. Data were collected from five diesel-powered HDDVs and varying driving conditions. For the single N3-type HDDV, the driving routes consisted of 20% urban roads, 25% rural roads, and 55% motorways. For the four M3-type HDDVs, the routes comprised 45% urban roads, 25% rural roads, and 30% motorways. Each vehicle operated an average of 5 days per week for approximately 2 h per day, yielding between 4000 and 16,000 data samples per month for model training. The findings showed that the RF model attained a strong average R^2^ of 0.706 along with low RMSE and MAE values of 0.0335 and 0.0199, respectively.

The study in [52] aimed to predict NO_*x*_ emissions under real-world driving conditions while incorporating driver behavior and vehicle dynamics. Temporal data were subjected to maximum difference segmentation and temporal feature transfer. Segmentation was carried out based on the maximum entropy principle, which divides time series data into subsegments of maximum variability to facilitate the application of transfer learning and address dynamic distributional changes in time series data. A distribution approximation method was proposed integrating a temporal fusion transformer Gated Recurrent Unit (GRU) and a back-propagation neural network. Within this processing framework, continuous time series data were partitioned into high-variability subsegments, which were then transferred for temporal feature learning. The dataset was collected from a diesel vehicle, while the OBD parameters included actual output torque percentage, engine oil temperature, aftertreatment downstream oxygen percentage, environmental temperature, aftertreatment waste mass flow rate, gas pedal position, and downstream NO_*x*_ concentration. After preprocessing, 1420 continuous data points were used for experimentation, with 1300 used for training and 120 for testing. The temporal fusion transformer GRU regressor predicted NO_*x*_ values with an MAE of 0.0545 and an RMSE of 0.0708.

A soft sensor approach for monitoring NO_*x*_ and particulate number (PN) emissions was proposed in [53]. Signal processing techniques included a genetic algorithm, GRU, LSTM, SVM, backpropagation neural network, stochastic gradient descent optimization, and GBDT. These algorithms were implemented locally in the vehicle using Python in order to develop soft sensors specifically designed for emission monitoring. The GRU model hyperparameters were optimized using a genetic algorithm to minimize training time and enhance performance. Experiments were conducted using diesel-powered HDTs, and the driving scenarios consisted of 20% urban, 25% suburban, and 55% highway conditions. A PEMS (specifically the OBS-ONE unit by HORIBA Ltd., Kyoto, Japan) was utilized, which includes emission analyzers, exhaust flow meters, OBD interface, environmental monitoring unit, and GPS. Thirteen OBD parameters strongly correlated with NO_*x*_ emissions were identified for model training, including engine coolant temperature, vehicle speed, mass air flow rate, ambient temperature, engine oil temperature, fuel rate, percent engine torque, load value, catalyst temperature, and several DPF-related variables. Different OBD parameters were found to be more suitable for PN prediction. Using the proposed soft sensor, the highest R^2^ for NO_*x*_ prediction was 0.9877 under highway conditions, while for PN emissions it reached 0.9260 in suburban conditions.

The study in [54] developed a model to detect and predict NO_*x*_ emissions, emphasizing the identification of high-emission patterns induced by unstable driving behavior. The proposed DL-based approach was built upon a custom Sequence-to-Sequence (Seq2Seq) neural network architecture. This approach consisted of a bidirectional GRU for embedding driving states, an attention mechanism for feature aggregation, and Incremental Tracking Loss (ITL) for enhancing sensitivity to high-emission episodes. The complete framework was implemented in PyTorch [55] and run on a Linux computing cluster. To this end, localized data collection and analysis were performed. Various OBD parameters were considered, including instantaneous speed, acceleration, engine cooling water temperature, and throttle pedal degree. Experiments are carried out across driving states, such as acceleration, deceleration, cruising, and idling. A total of 12,628 samples were collected from 21 driving trajectories using a HDDV, primarily operating on highways. Based on the results, the proposed method significantly outperformed existing techniques, achieving RMSE reductions of 62% overall and 73.6% for high-emission cases. Additionally, the proposed model attained an MAE of 4.406 overall and MAPE of 1.568% for high-emission samples.

### 4.2. Monitoring and Prediction of CO_2_ Emissions

The work in [56] presented an AI-based module based on TinyML for estimating CO_2_ emissions from vehicles. Various ML-based and signal processing techniques were employed, including linear regression, supervised and unsupervised learning, the Typicality and Eccentricity Data Analysis (TEDA) algorithm, and digital twins via the Simulation of Urban Mobility (SUMO) traffic simulator. These techniques were used for CO_2_ emissions estimation, outlier detection, and pollution simulation, and were deployed in the cloud using Python 3.10, the Traci library, and LazyPredictor. OBD data were collected using the Torque Pro mobile application, which facilitated communication between the vehicle’s OBD system and cloud-based tools. The recorded parameters included MAP, MAF, and data for calculating the air–fuel ratio, speed, and acceleration. A single driver operating a flexible hybrid vehicle (Nissan Kicks) was involved in the data collection, which was conducted in the city of Natal, Brazil along a 13 km urban route. In addition, two fuel scenarios, gasoline and ethanol, were tested over ten trips for each fuel type. The SUMO simulator provided emission estimates which were compared with results calculated from real OBD sensor data. Based on the results, the simulated ethanol model yielded an MAE of 0.2334 and an RMSE of 0.3624, both significantly lower than those for gasoline.

An AI-driven computational model for CO_2_ emissions was proposed in [57] utilizing linear regression, RF, and gradient boosting techniques for emission modeling in a Euro 6 start–stop vehicle. Acceleration and road gradient were key input features. The model evaluation was based on MSE and R^2^ as well as visual validation via emission maps, instantaneous emission profiles, and residual plots. OBD parameters such as velocity, acceleration, fuel consumption, CO_2_ emissions, humidity, air temperature, GPS coordinates, and altitude were sampled, yielding over 3000 data records. These were integrated with data from the HORIBA OBS-2200 PEMS and road gradient measurements. The test vehicle was a 2018 Euro 6 diesel vehicle featuring a 1560 cm³ engine delivering 88 kW at 3500 RPM, a manual 6-speed transmission, common rail injection, and an exhaust aftertreatment system comprising DPF, SCR, and diesel oxidation catalyst (DOC). The driving conditions spanned urban, rural, and highway segments, with the experiments conducted over 111 km in Rzeszów, Poland. The gradient boosting technique achieved the best test MSE of 0.3551 and R^2^ of 0.7423.

The authors of [58] proposed a twofold approach involving the development of a prognostic model for trip-fixed CO_2_ emissions based on observed data followed by validation of the capability to estimate emissions in alignment with EU regulatory standards using ML. Specifically, the Extreme Gradient Boosting (XGBoost) supervised ML algorithm was utilized to predict real-road CO_2_ emissions from HDDVs and ultra-heavy-duty vehicles (UHDVs). The prediction methodology was executed locally within the vehicle during testing and validated using real-world data. Collected OBD parameters included engine speed, throttle position, fuel rate, and vehicle speed, all gathered across multiple trips. OBD-II data were combined with emission data measured via PEMS. Furthermore, the driving experiments were conducted in South Korea using diesel-powered vehicles complying with Korean RDE regulations: a 3.5-ton HDDV, and a 25-ton UHDV. The methodology supported emission testing and regulatory evaluation for Euro 6 compliance, particularly under Euro 6 Step C and Step D standards, which include segments characterized by traffic congestion. Evaluation metrics included R^2^, RMSE, and MAPE. The validation results demonstrated high accuracy, with R^2^ values of 0.942 for HDDVs and 0.981 for UHDVs.

In [59], the authors assessed the effectiveness of DL models in estimating CO_2_ emissions across both local and roadway environments. This study employed signal processing techniques such as Deep Neural Networks (DNNs), Convolutional Neural Networks (CNNs), and LSTM networks to model vehicular CO_2_ emissions using OBD-II data. The experimentation emphasized feature selection and the tuning of internal LSTM parameters such as network width and layer depth. The driving experiments involved two petrol-powered vehicles: a Renault (1.0 L, 76 cv) and a Hyundai (1.6 L, 122 cv). Naturalistic experiments were carried out with ten drivers using the first vehicle, while controlled experiments were conducted with four drivers using the second vehicle. The relevant OBD-II data encompassed vehicle speed, engine RPM, mileage, fuel flow, throttle position, and acceleration, and are publicly available through the Rettore dataset (https://www.rettore.com.br/public_data/vehicular-trace/(accessed on 30 April 2025)). These OBD-II data were augmented with additional information on trip characteristics, driver gender, and age. The proposed LSTM model achieved an MAE of 6.16, MSE of 86.45, and RMSE of 9.30 on the original test data, outperforming the DNN (MAE: 47.53, RMSE: 64.87) and CNN (MAE: 17.98, RMSE: 17.82). When tested with noisy data, the LSTM model maintained high robustness with an MAE of 11.36 and RMSE of 13.15 compared to DNN (MAE: 89.67, RMSE: 124.51) and CNN (MAE: 45.48, RMSE: 37.46).

**Table 4:** Overview of ML-based approaches for monitoring and prediction of CO_2_ emissions.

Reference	Objective	ML Models	Driving Environment	Type of Vehicle	OBD or OBD-Derived Parameters	Performance Metrics
Andrade et al., 2024 [56]	Estimation of CO_2_ emissions and comparison of emissions from gasoline and ethanol	Linear regression, supervised/unsupervised learning	The route was approximately 13 km in urban areas with paved and asphalt sections	Flexible hybrid vehicle (gasoline, ethanol), Nissan Kicks	MAP, MAF for calculation of air-fuel ratio, speed and acceleration	MAE, RMSE
Madziel et al., 2023 [57]	Development of a computational model for CO_2_ emissions	Linear regression, RF, gradient boosting	Characterization by segments meditating on urban, rural and highway environments	Euro 6-compliant vehicle equipped with a diesel engine	Velocity, vehicle acceleration, fuel consumption, CO_2_ emission, humidity, air temperature, latitude, longitude, altitude	MSE, R^2^
Moon et al., 2024 [58]	Prognostic model for trip-based CO_2_ emissions using observed data	XGBoost	Routes reflected real-road conditions and included congestion segments	3.5-ton HDDV and 25-ton UHDV	Engine speed, throttle position, derived engine torque, engine coolant temperature, fuel rate, vehicle speed	R^2^, RMSE, MAPE
Singh et al., 2023 [59]	Accurate CO_2_ estimation	DNN, deep CNN, LSTM network	Naturalistic experiments and controlled experiments	Petrol-powered Renault and Hyundai vehicles	Speed, engine RPM, mileage, fuel flow, throttle, acceleration	MAE, MSE, RMSE

### 4.3. Monitoring and Prediction of Multiple Pollutants and Other Emissions

The objective of the work in [60] was to predict NO_*x*_ and CO_2_ emissions from HDDVs by leveraging a wide range of techniques such as LSTM networks, back-propagation neural networks, particle swarm optimization (PSO), and weighted concatenation. These techniques aimed to extract features from OBD-II indicators, capture temporal dependencies, optimize feature weights, and perform feature fusion. This work involved fifteen N2-type vehicles (mass ranging from 3500 to 12,000 kg) and eight N3-type vehicles (mass exceeding 12,000 kg), all equipped with conventional internal combustion engines and operating under complex real-time driving conditions. The OBD parameters included the instantaneous concentration of NO_*x*_ in exhaust gases, fuel flow rate, vehicle speed, atmospheric pressure, DPF pressure difference, cumulative mileage, reagent residual quantity, and SCR inlet/outlet temperatures. Actual NO_*x*_ and CO_2_ emission values were used to validate the predictions derived from the OBD-recorded parameters. The proposed model integrating PSO-optimized multimodal feature fusion with LSTM demonstrated significant improvements over baseline LSTM models. For NO_*x*_ prediction, this model achieved an MSE of 66.496 compared to 109.712 for the baseline LSTM model, representing a 39.082% reduction in error. For CO_2_ prediction, this model reduced the MSE by 82.647% compared to the baseline, highlighting its superior accuracy. Fuel flow rate was identified as the most influential parameter for both NO_*x*_ and CO_2_ emissions, with weights of −0.828 and 0.882, respectively, followed by SCR-related indicators for NO_*x*_ and engine parameters for CO_2_.

The authors of [61] investigated elevated NO_*x*_ and CO_2_ emissions from HDDVs under various engine states and road grades (uphill, downhill, flat). Using an online platform, they applied the RF, XGBoost, elastic net regression, feature importance modeling, SHAP, Kendall’s Tau, and k-means ML techniques for correlation analysis, clustering, and local emission prediction. OBD-II data (engine/tailpipe NO_*x*_, acceleration, accelerator pedal position, engine net output torque, air–fuel ratio, and engine rotation speed) were enriched with Digital Elevation Model (DEM) and GPS data, yielding 13,608 samples over a 653 km freeway route. The HDDV had an exhaust gas recirculation (EGR)-equipped engine, and was used for 262 short trips categorized by road grade. Results showed that aggressive driving, large pedal openings, and high loads raised NO_*x*_ and CO_2_ emissions. SHAP analysis indicated that rich/lean AFR increased CO_2_, while lean states elevated NO_*x*_ due to higher temperatures and lower SCR efficiency. Uphill segments saw the highest emission rates (100 μg/s NO_*x*_, 39.59 g/s CO_2_), compared to 23.29 g/s CO_2_ downhill. RF outperformed XGBoost in NO_*x*_ prediction (MSE: 66.496 vs. 109.712) and reduced the CO_2_ prediction error by 82.647%, although the exact MSE was unspecified.

The study in [62] focused on reducing carbon emissions by analyzing HDT operations based on load status, trip characteristics, and start–stop cycles. OBD-II data from 3792 HDTs (cargo, dump, and tractor trucks) were collected through a remote online monitoring platform. Using big data mining and computational techniques (e.g., dwell detection, load identification, and origin–destination (OD) trip information calculation), this study leveraged parameters such as velocity, fuel flow, and GPS data and used Kepler GL for visualization. Cargo trucks, dump trucks, and tractor trucks exhibited empty-load driving rates of 33.01%, 33.58%, and 31.71%, respectively. Average OD distances were 110 km, 95 km, and 195 km, with speeds of 23 km/h, 16 km/h, and 40 km/h and start–stop counts of 2.8, 3.9, and 4.9 per trip, respectively. Tractor trucks operated both intra-city and long-distance routes; cargo trucks stayed within cities, while dump trucks were concentrated in major regional cities. Data preprocessing removed 14.1% of the samples, with gaps (2–300 s) filled to ensure continuity. Results showed that OBD-based analysis can enhance truckload planning and driving behavior, enabling faster and more precise emission reductions than manual methods.

The study in [63] addressed the quantification and prediction of urban black carbon (BC) emissions from light-duty gasoline vehicles (LDGVs) using ML and real-world emission data. BC emissions were measured using a PEMS and predicted with models built from OBD-II data collected from five LDGVs. The vehicles were compliant with China VI standards, included both gasoline direct injection and port fuel injection engine types, and were tested across five urban road categories. ML algorithms (RF, GBDT, XGBoost, Light Gradient Boosting Machine (LightGBM), and Multiple Linear Regression (MLR)) were developed using Python 3.8 and scikit-learn 1.3.0 to predict both instantaneous and cumulative BC emissions. The key input features included fuel consumption, engine RPM, engine load, throttle position, and vehicle speed. Among these, engine speed and load showed the strongest correlation with BC emissions (R^2^ = 0.5–0.9). Based on the results, the RF model consistently achieved the highest predictive accuracy (R^2^ > 0.6 for all vehicles), followed closely by LightGBM and XGBoost, while MLR showed the weakest performance. For example, in one test case RF achieved an MAE of $1.90\times 10^{-4}$ mg/s and R^2^ = 0.62, while MLR yielded an MAE of $2.45\times 10^{-4}$ mg/s and R^2^ = 0.43.

A parametric model for estimating the impact of gear position on pollutant emissions (CO_2_, CO, NO_*x*_, and hydrocarbons (HC)) was proposed in [64] with the aim of developing a low-cost and reliable emission estimator for light-duty vehicles. The methodology leveraged GPS data and ML techniques to eliminate dependence on expensive tools such as PEMS. Gear estimation was achieved through K-means clustering and classification trees, while RF was used to determine predictor importance and ANNs were trained to estimate pollutant emissions. Inputs included GPS and OBD data such as vehicle speed, engine RPM, fuel flow, and calculated driving forces (e.g., aerodynamic, rolling, and gravitational resistances). Experiments were conducted using three gasoline-powered vehicles, a sedan (1.4 L), an SUV (2.0 L), and a pickup (2.4 L), traveling over urban, rural, and highway segments. The training dataset was drawn from real driving data covering 324, 300, and 316 km for the sedan, SUV, and pickup, respectively. ANN performance was evaluated using R^2^ and MSE, achieving high accuracy: CO_2_ (R^2^ = 0.7358, MSE = 0.2183), CO (R^2^ = 0.8616, MSE = 0.0032), NO_*x*_ (R^2^ = 0.7992, MSE = $1.647\times 10^{-5}$), and HC (R^2^ = 0.8920, MSE = $7.974\times 10^{-8}$).

The work in [65] aimed to enhance urban air quality by predicting vehicle emissions (NO, NO_2_, CO, CO_2_, and total hydrocarbons (THC)) using advanced ML models, notably a parallel attention-based LSTM (PA-LSTM). When compared with ARIMA, SVR, RNN, LSTM, attention-based variants, and dual-stage attention-based RNN (DA-RNN), PA-LSTM consistently outperformed alternatives. Implemented in PyTorch, the models used OBD-II (i.e., vehicle speed, engine power, throttle voltage, RPM, load, wheel force, oil temperature, and ambient temperature), and PEMS data (i.e., exhaust flow rate, exhaust temperature, and air/fuel ratio). Experiments were conducted on a diesel FAW medium-duty truck (CHINA V, 2.2 L engine, 15,700 kg) in real-world conditions (Beijing) and lab settings (Tianjin). For NO prediction, PA-LSTM achieved RMSE/MAE of 18.003/11.873 ppm (road) and 23.542/14.383 ppm (bench), outperforming ARIMA (RMSE: 46.328, MAE: 27.197) and standard LSTM (RMSE: 35.732 ppm, MAE: 22.173 ppm for road test). It also reached an MAPE of 9.945% (road) and 11.678% (bench), with R^2^ values of 97.58% and 96.47%, respectively, indicating high prediction accuracy. PA-LSTM also excelled in early warning of high emissions, achieving an average recognition accuracy of 87% across NO, NO_2_, CO, and THC compared to 85% for DA-RNN and 69.25% for ARIMA.

The study in [66] aimed to model exhaust emission from older vehicles in order to aid environmental monitoring and policy development, focusing on ${\mathrm{CO}}_{2}$, CO, THC, and NO_*x*_ under cold and warm engine conditions. ML techniques included regression-based predictive modeling and spectral clustering, which were employed to analyze emissions from a Euro 2 class passenger car (1998 model, 1598 cm^3^ gasoline-powered spark-ignition engine, 88 kW, 1230 kg). Data were collected using a PEMS and OBD-II interface, capturing parameters such as vehicle speed and acceleration during a 40 km test route in Rzeszów, Poland that included urban, expressway, and motorway segments. Spectral clustering based on speed and acceleration yielded four clusters for cold engine states and two for warm states. Regression models (linear, polynomial, Lasso, Ridge, DT, RF, SVM, gradient boosting) were trained in Python (Google Colab) and evaluated via 5-fold cross-validation. Under cold conditions, RF performed best for THC (MSE: 0.00003, R^2^: 0.756), polynomial regression for ${\mathrm{CO}}_{2}$ (MSE: 0.00319, R^2^: 0.926), gradient boosting for CO (MSE: 0.00288, R^2^: 0.485), and polynomial for NO_*x*_ (MSE: 0.00005, R^2^: 0.602). For warm engines, polynomial regression again led for ${\mathrm{CO}}_{2}$ (MSE: 0.00219, R^2^: 0.954), while gradient boosting was best for THC (MSE: 0.00002, R^2^: 0.665). Despite strong results for ${\mathrm{CO}}_{2}$ and THC, lower R^2^ values for CO and NO_*x*_ reflect challenges from catalytic converter effects and combustion variability.

**Table 5:** Overview of ML-based approaches for monitoring and prediction of multiple pollutants and other emissions.

Reference	Objective	ML Models	Driving Environment	Type of Vehicle	OBD or OBD-Derived Parameters	Performance Metrics
Li et al., 2024 [60]	Prediction of NO_*x*_ and CO_2_ emission	LSTM network, back-propagation neural network, PSO	Complex real-time driving conditions in several cities	HDDVs with conventional internal combustion engines	Instantaneous concentration of NO_*x*_ in the exhaust gas, fuel flow rate, friction torque, DPF pressure difference, cumulative mileage, reagent residual quantity, SCR inlet/outlet temperature	MSE
Xie et al., 2024 [61]	Analysis of the attributes of high NO_*x*_ and CO_2_ emission in connection to the vehicle states of running and engine operation	K-means clustering, RF, XGBoost, elastic net	Uphill, flat, and downhill road segments	HDDV with an engine equipped with EGR valves	Engine output NO_*x*_, tailpipe NO_*x*_, engine speed, engine output power, accelerator pedal opening, air fuel ratio, rotstion speed, accelerator pedal opening	MSE
Wang et al., 2024 [62]	Reduction of carbon emissions	Gaussian mixture along with big data mining	urban areas, freeways, and construction sites	Cargo trucks, dump trucks, tractor trucks all with diesel engines	Velocity, fuel flow rate, latitude, longitude	No need for evaluation since an operation analysis was performed
Wang et al., 2024 [63]	Assessment and prediction of instantaneous and total BC emissions	RF, GBDT, XGBoost, LGBM, MLR	Different urban road types including branch roads, sub-arterial ways, arterial, express roads and freeways	Light-duty vehicles with gasoline direct or port injection engines	Fuel consumption, engine RPM, engine load, throttle position, engine speed, vehicle speed	RMSE, MAE, R^2^
Rivera-Campoverde et al., 2024 [64]	Estimation of pollutant emissions (CO_2_, CO, NO_*x*_, and HC)	K-means clustering, classification trees, RF, ANNs	Urban, rural, and highway driving conditions	Light vehicles with gasoline engines: Sedan (1.4 L), SUV (2.0 L), Pickup (2.4 L)	Engine speed, vehicle speed, fuel flow, pollutant data (CO_2_, CO, NO_*x*_, HC)	R^2^, MSE
Xi et al., 2021 [65]	Prediction of NO, NO_2_, CO, CO_2_, and THC in urban areas	ARIMA model, SVR, dual-stage attention-based RNN, parallel attention-based LSTM network	Actual road conditions and bench condition in controlled experiment	Diesel vehicle of type FAW-Medium Duty Truck	Vehicle speed, engine power, throttle voltage, RPM, load, wheel force, oil temperature and ambient temperature	RMSE, MAE, MAPE, R^2^
Madziel et al., 2024 [66]	Analysis of emissions (${\mathrm{CO}}_{2}$, CO, THC, and NO_*x*_) under cold and warm engine conditions	Polynomial regression, RF, gradient boosting, SVM, and spectral clustering	A 40 km test route covering urban, expressway, and motorway segments	Euro 2 class car with a gasoline-powered internal combustion engine	Speed, acceleration, and emissions of CO_2_, CO, THC, and NO_*x*_	R^2^, MSE

## 5. Automotive Behavior and Driver Analysis

Driving behavior is influenced by external factors (e.g., itinerary, road, weather) and driver-specific traits (e.g., personality, temperament, and idiosyncrasies) as well as time-dependent conditions such as fatigue and stress. Aspects such as cautiousness, patience, aggressiveness, and conservativeness are reflected in driving behavior as well as in high-level driving events, e.g., acceleration, deceleration, engine revving, etc. Understanding driving behavior enhances safety, convenience, energy efficiency, and emission reduction; however, traditional OBD-II systems lack the capability to analyze driver behavior beyond basic metrics (e.g., speed violations) and rely on manual interpretation or simple heuristics, which cannot capture nuanced patterns such as aggressive acceleration or risky driving habits. This section reviews recent works on safety, driving behavior profiling, and driver identification based on behavioral patterns, with each domain respectively outlined in Table 6, Table 7 and Table 8.

### 5.1. Safety Monitoring

The work in [67] proposed a pedestrian risk prediction mechanism to enhance safety by estimating the number of pedestrians intending to cross a street based on vehicle-mounted camera data. The system utilized the PIE dataset, consisting of six hours of dashboard camera footage recorded in Toronto, along with vehicle speed data from the OBD-II module. A real-time alert was generated if the vehicle exceeded a configurable speed threshold while pedestrians were detected. Pedestrian intent was modeled as a binary classification task by leveraging video-based motion analysis and pedestrian-vehicle interaction features. To avoid performance saturation in the DNNs, a residual neural network architecture was employed. The system comprised four modules: (i) extraction of joint pedestrian-environment interaction features from video; (ii) monitoring pedestrian-vehicle and vehicle-vehicle interactions; (iii) modeling pedestrian movement via a GRU with 256 hidden units; and (iv) prediction of intent using the binary cross-entropy loss. Based on the results, the model achieved 83% accuracy on the PIE dataset.

In [68], the authors proposed an ML-based system for direct post-crash severity assessment by classifying incidents as slight, serious, or fatal. The goal was to aid emergency response systems in prompt assessing and improving assistance provided. This study leveraged a large-scale dataset from the UK Department for Transport covering over 2 million accident cases (2005–2017). The dataset contained 34 features, including road conditions, vehicle parameters (some from OBD systems), and environmental factors. After removing features with high null values, the dataset was preprocessed via data cleaning, feature selection, oversampling to mitigate class imbalance, and one-hot encoding for categorical variables. Four ML models were trained and evaluated: DT, RF, XGBoost, and LR. RF achieved the highest performance with 95% accuracy, while XGBoost performed poorly (52%) due to its sensitivity to class imbalance. The precision, recall, and F1-score were reported for all models across all severity classes. This system’s practical value lies in integrating vehicle-side data for smart city safety infrastructure. The incorporation of IoT and image processing systems may further contribute to real-time crash detection and severity estimation, while the use of live vehicular telemetry or camera feeds may further contribute to predictive accuracy and decision support in emergency response systems.

The work in [69] presented a multi-stage accident detection and severity assessment system tailored for powered two-wheelers (PTWs) and capable of addressing their inherently unstable dynamics. This system consisted of three main modules: critical event detection, accident confirmation, and severity assessment, which were combined with accelerometer-based OBD data and physiological data from the rider. Accelerometers placed on both the PTW and the rider’s helmet sampled tri-axial data at 45 Hz, enabling state classification into normal, fall-like, and fall categories. To preprocess and label the pre-existing unlabeled dataset [70], K-means clustering was applied, achieving 71.24% clustering accuracy. Several classifiers (naive Bayes, ANN, RNN, and a modified DT with tanh entropy) were evaluated for state classification. The modified J48 DT achieved 100% accuracy, precision, and recall across all classes along with significantly reduced convergence time (1.31 s). To confirm falls and reduce false positives from skidding or leaning, an Adaptive Sequence Window (ASW) algorithm was introduced, which adjusted detection sensitivity based on real-time state transitions. Accident severity was determined using a rule-based logic system incorporating fall states and the rider’s dynamically estimated pulse rate baseline. This framework demonstrated exceptional real-time detection capabilities with minimal hardware and high classification fidelity, making it ideal for lightweight and low-cost PTW safety systems.

**Table 6:** Overview of ML-based approaches for safety monitoring.

Reference	Objective	ML Models	Driving Environment	Type of Vehicle	OBD or OBD-Derived Parameters	Performance Metrics
Arzhmand et al., 2023 [67]	Enhancement of real-time safety aspects, understanding street-crossing pedestrian context	Residual Neural Networks, conditional statements	6 h of video from traffic scenes in Toronto, recorded by dashboard camera (based on PIE dataset)	Not mentioned	Speed	Accuracy, precision, area under the curve (AUC), recall, F1 score
Koley et al., 2022 [68]	Estimation of vehicular crash severity	DT, RF classifier, XGBoost, LR	Based on UK driving accident dataset involving vehicles (2005–2017), including mainly urban areas	Vehicles are described in terms of age and capacity	Speed for severity estimation (not used in this study)	Accuracy on the estimation of the severity of a class accident
Mahariba et al., 2022 [69]	Accident detection	Naïve Bayes, DT, ANN, RNN, J48	Data retrieved from PTWs, based on predefined crash scenarios (fall in a curve or slippery straight maneuver) performed by professional riders	Two-wheelers; professional riders; 97.2 cc engine with electronic ignition and double cradle frame	OBD unit with two accelerometers (one on the vehicle, one on the rider’s helmet)	Precision, recall, F-measure, Matthew’s correlation coefficient, ROC curve, precision–recall curve
Ahmad et al., 2024 [71]	Detect and evaluate DDD	RNN (DDD-DL)	Custom dataset using OBD-II data converted into time series with timestamps; drowsiness identified via video signal	Not explicitly mentioned; in-car camera used	RPM, throttle position, steering torque, combined with in-car video	Accuracy, precision, recall, and F1-score across driver states
Kumar et al., 2022 [72]	Incident detection and evidence integrity protection	Rule-based comparison with thresholds	Not mentioned	Not mentioned	Speed, RPM, torque, throttle position, relative throttle position, engine load	Correctly vs. erroneously identified incidents

The work in [71] developed a hybrid Driver Drowsiness Detection (DDD) system integrating vehicle telemetry data from OBD-II with video-based behavioral cues. Their approach aimed to address limitations in accuracy and real-time applicability typically associated with single-modality systems. The custom dataset used in this study consisted of time-synchronized OBD-II parameters (vehicle speed, RPM, throttle position, and steering torque) alongside facial video recordings of the driver captured during real driving sessions. Video data were labeled using a pretrained transfer learning model to classify driver states (e.g., “drowsy” vs. “normal”), then these labels were aligned with the corresponding time series vehicle data to build a supervised dataset. The proposed DL-based model, called DDD-DL, was based on an RNN architecture incorporating two stacked LSTM layers followed by dropout and batch normalization layers to mitigate overfitting and improve generalization. Feature vectors were padded to uniform sequence lengths (7000 samples) and the dataset was balanced through upsampling. The model was trained using the Adam optimizer and binary cross-entropy loss for 200 epochs with a batch size of 64. Evaluation yielded an overall accuracy of 81.95%, outperforming the baseline SVM (72.18%) and LR (60.90%) models. The DDD-DL model achieved class-specific F1-scores of 82.61% (drowsy) and 81.25% (normal), indicating strong discriminative ability.

In [72], an innovative framework was proposed that integrates real-time OBD-II data collection with decentralized blockchain-based storage to detect vehicular incidents and analyze driver behavior. The system utilized an OBD-II dongle connected to an edge device (Jetson Nano, NVIDIA, Santa Clara, CA, USA) via Wi-Fi, which was used to collect vehicle telemetry data such as speed, RPM, GPS, and video footage. Data preprocessing included conversion from Comma Separated Values (CSV) to JavaScript Object Notation (JSON) using Apache Kafka, followed by streaming to the edge for real-time analytics using PySpark. Incident detection was based on a thresholding mechanism applied to acceleration values along the x, y, and z axes, where deviations beyond ±3 m/s^2^ indicated abnormal behavior such as hard braking, swerving, or encountering road anomalies. When such events were detected, the system captured a 30-s buffer of sensor and video data, uploaded it to InterPlanetary File System (IPFS), and stored the corresponding hash along with vehicle ID and geolocation on the Ethereum blockchain via smart contract. This architecture ensures data immutability and integrity for forensic and insurance applications. The system achieved an incident detection accuracy of 82% on a dataset containing 5300 records, including 247 labeled events. Additionally, smart contract execution incurred a minimal gas cost (ETH 0.034806 for deployment and ETH 0.000845 per write).

### 5.2. Driving Behavior Profiling

In [73], the authors investigated driving styles in Mobility-as-a-Service (MaaS) scenarios, motivated by the potential to improve driving quality, environmental efficiency, and passenger experience. Two datasets were utilized: (i) meta-information on the vehicle (e.g., manufacture year, model, fuel type) along with user profiles (e.g., age, gender, preferences) collected via questionnaires; and (ii) trip data, including OBD-II records, smartphone-based context (e.g., location, signal strength, sensors), and web-sourced traffic/weather information (TomTom Application Programming Interface (API)). Feedback from drivers and passengers was also incorporated. Moreover, K-means clustering with the elbow method was applied to the automotive data, revealing five distinct driving behavior clusters, with speed and acceleration as key factors. Eco-efficiency analysis identified optimal behavioral patterns (e.g., moderate acceleration, smooth deceleration, and optimal RPM), while correlation analysis integrated human input. The findings showed that sudden acceleration/deceleration negatively affects passenger perception, which generally remains low regarding driving efficiency.

In [74], the authors proposed a privacy-preserving system for detecting unsafe driving behaviors in HEVs, employing LSTM, GRU, and their bidirectional variants (BiLSTM and BiGRU). The architecture integrated vehicle-mounted OBD-II interfaces with an ECU to collect driving data without using privacy-sensitive features such as GPS or driver images. The system simulated four driving behaviors on a controlled track: safe, aggressive acceleration, sharp turning, and hard braking. Six drivers operated a Toyota Prius V2ZR-FXE. Over 1.5 million real-time data points were initially recorded, then refined to 76,948 labeled samples. The input features included battery voltage, throttle position, MAF, steering angle, wheel speed, and lateral acceleration. Data were preprocessed through min–max normalization and fed into multi-class classifiers trained using Softmax- and ReLU-activated hidden layers, with dropout layers added to mitigate overfitting. The GRU-based model trained for 1000 epochs achieved 97.5% accuracy, a Kappa score of 0.965, and a minimal loss of 0.081, outperforming LSTM and other evaluated models. Confusion matrix analysis revealed high class-specific precision, with aggressive acceleration being the most accurately predicted. The system delivers real-time alerts to the vehicle dashboard via secure web APIs, offering practical deployment potential. This approach balances effective behavioral classification with data privacy by omitting location-based or biometric inputs and instead relying on in-vehicle kinematic parameters.

The work in [75] introduced a semi-supervised learning approach for driving style recognition using a Gaussian Mixture Model (GMM), aiming to reduce reliance on labeled data while maintaining high classification accuracy. Real-world vehicle data were collected via OBD-II, extracting 22 driving style features (e.g., speed, jerk, statistical descriptors). To address multicollinearity and reduce computational complexity, Kernel Principal Component Analysis (KPCA) was applied, yielding six principal components that together explained over 70% of the variance in the dataset. The semi-supervised GMM leveraged both labeled and unlabeled data. Initial parameters for the model were the means, covariance matrices, and mixture weights, which were generated from labeled samples. The Expectation-Maximization algorithm was used to iteratively refine these parameters by using likelihood maximization and posterior probability estimation to infer class memberships for the unlabeled samples. The driving styles were categorized into three classes: aggressive, normal, and conservative. The authors evaluated the model using driving data segmented into time windows of 10 s, 30 s, and 50 s. The 30 s window was determined to be optimal, balancing recognition timeliness with stability. When compared against standard classifiers (e.g., k-means, SVM, unsupervised GMM), the proposed semi-supervised GMM achieved the highest performance, with an accuracy of 92%, macro precision of 92.2%, and macro F1-score of 92.15%.

The work in [76] presented a rule-based framework for identifying driving events and analyzing correlations among engine operational parameters using OBD-II time series data. Focusing on core metrics such as RPM, throttle position, manifold absolute pressure, and engine load, the authors analyzed data from both an open dataset (KIA, automatic transmission) and a custom dataset (Toyota iQ, manual transmission) under three key driving modes: idle, cruising, and acceleration. The authors developed and validated simple yet effective rules to detect idle states, gear changes, and accelerations, achieving high accuracy (95% for gear changes and 91% for acceleration in automatic transmissions; 88.5% for gear changes and 82% for acceleration in manual transmissions). These rules were used to quantitatively characterize driving style and are inherently transparent, offering clear interpretability. To explore parameter relationships, the authors applied the catch22 time series feature set, revealing strong correlations between engine load, throttle position, and manifold pressure during acceleration, while idle mode showed weaker associations. Such rule-based approaches may contribute to the design of XAI in vehicle diagnostics, ensuring interpretable intermediate representations and fusing expert knowledge with data-driven models.

The authors of [77] investigated the classification of safe versus unsafe driving behaviors using a suite of gradient-boosting algorithms applied to in-vehicle sensor data obtained via the OBD-II interface. In this context, the publicly available OBD-II dataset [78] was used, comprising 94,380 time-stamped entries recorded at 1-s intervals over 26 h of driving conducted by ten drivers across urban, highway, and parking environments. A total of 51 parameters were collected, including key engine and vehicle dynamics signals such as throttle position, engine torque, speed, steering angle, gear state, and brake pressure. Following feature selection using the SelectFromModel learning method, several boosting classifiers—CatBoost, AdaBoost, LightGBM, Gradient Boosting, and XGBoost—were trained to perform binary classification of driving behavior. The models were trained with an 80/20 split for training and testing and their performance was evaluated using accuracy, precision, recall, F1-score, ROC curves, and confusion matrices. LightGBM emerged as the most effective classifier, achieving the highest accuracy of 99.68% and with precision, recall, and F1-score values all near or exceeding 0.996. CatBoost and XGBoost also demonstrated strong predictive capabilities (accuracy > 99%).

**Table 7:** Overview of ML-based approaches for driving behavior profiling.

Reference	Objective	ML Models	Driving Environment	Type of Vehicle	OBD or OBD-Derived Parameters	Performance Metrics
Gace et al., 2023 [73]	Analyze eco-efficiency and driving behavior to promote sustainable transportation practices.	K-Means Clustering and Eco-Efficiency Analysis.	Primarily urban and suburban driving in Zagreb, Croatia.	Vehicles included gasoline, diesel, and hybrid powertrains.	Vehicle speed, RPM, accelerator pedal position, and engine load.	Eco-efficiency Index (Ieco), acceleration/deceleration thresholds, and idling percentages
Lee et al., 2023 [74]	Driving behavior analysis, provision of feedback to the driver	LSTM networks, GRUs and adaptations (BiLSTM and BiGRU)	Six drivers using a Toyota Prius V2ZR-FXE	Hybrid and HEV	Battery output voltage, vehicle speed, calculated load, MAF, coolant temperature, throttle voltage, yaw, steering angle, lateral acceleration, forward and rearward acceleration, vehicle front/rear left/right wheel speed	Accuracy, recall, precision, F1-score, Kappa score as a statistical measure to evaluate the consistency between evaluators
Song et al., 2023 [75]	Recognition of driving style and classify driving style (normal, aggressive, conservative)	Semi-supervised Gaussian mixture, kernel PCA (nonlinear mapping into six components)	Follow the precedent car and use time windows for recognition and prediction	Not mentioned explicitly	Velocity, acceleration, jerk (22 parameters)	Accuracy rate, macro precision rate, macro recall rate, and macro F1 comparing 4 classification methods
Rimpas et al., 2022 [76]	Interpret OBD-II parameters into meaningful events, correlate with fuel consumption and characterize driving style	Rule-based approach for event interpretation and fuel consumption	Korean dataset	Short-term fuel trim, MAP, absolute throttle position, rpm, calculated engine load, ECT, speed, and catalytic converter temperature	Accuracy of event interpretation	Gear change identification accuracy, acceleration identification accuracy, and idle identification accuracy
Divyasri et al., 2024 [77]	Driving behavior classification into safe or unsafe mode	Boosting algorithms including CatBoost, AdaBoost, LightGBM, GradientBoost, and XGBoost	Korean dataset conditions: urban and suburban environment, ten drivers	Korean dataset vehicles	Engine speed, engine torque, throttle position, vehicle speed, and steering wheel angle	ROC analysis, Accuracy, Precision, Recall, F1-score, and confusion matrices
Kumar et al., 2022 [79]	Classify and analyze driving behavior	Transformation of OBD-II primary data into secondary data (e.g., revving, stability, etc). SVM, AdaBoost, and RF	Korean dataset conditions: urban and suburban environment, ten drivers	Korean dataset vehicles	Fifty parameters, including speed, motor RPM, paddle position, calculated engine load	Accuracy of classification into ten classes

The work in [79] presented a driver behavior classification framework using OBD-II data and supervised ML algorithms (SVM, AdaBoost, and RF). The system captured over 50 vehicle parameters through the ELM327 Bluetooth-enabled OBD-II interface, covering metrics such as engine speed, throttle position, brake application, gear engagement, and steering behavior. Data were collected from ten drivers navigating a 46 km route under mixed traffic conditions, yielding a dataset of over 65,000 records. To enhance interpretability and classification accuracy, the authors derived eleven behavioral indicators, including average fuel consumption, engine idling time, high-speed braking events, speed stability, and gear usage, transforming raw sensor data into secondary features. Each driver was assigned a behavior score based on the cumulative influence of these indicators and classified into one of ten ranked categories, from “Poorest” to “Excellent”. Visualization tools (e.g., correlation matrices and feature plots) were employed to analyze interdependencies among parameters. The ML models were trained on 70% of the dataset and evaluated on the remaining 30%. The RF model achieved perfect classification accuracy (100%), while SVM and AdaBoost yielded 99% each. The models showed strong potential for real-time driver assessment in driver coaching, insurance premium estimation, and vehicle security systems.

### 5.3. Driver Identification

The work in [80] explored driver identification using multivariate time series data from OBD-II sensors, proposing two DL models: Bidirectional LSTM with Attention (BiLSTM-A) and a Modified Time Series Transformer (MTST). Both captured bidirectional driving behavior, including reaction and anticipation, by using different mechanisms: BiLSTM-A combines stacked LSTM layers with a custom attention layer, while MTST employs self-attention and positional encoding to model long-range dependencies without recurrence. Experiments were conducted on the public dataset from [78], which includes OBD-II recordings from ten drivers navigating the same urban track. The models were trained and evaluated using a 5-fold cross-validation strategy with a 10% buffer to prevent temporal leakage. Driver classification was performed using two approaches: single-segment prediction (60 s) and time-shift ensembles aggregating predictions over multiple shifted segments (up to 460 s). When using single segments, BiLSTM-A and MTST achieved 55% and 73% accuracy, respectively. When applying time-shift ensembles, performance significantly improved to 92% for BiLSTM-A and 97% for MTST. This work also assessed a reduced feature set limited to propulsion-system-independent attributes as well as scenarios for BEVs and alternative propulsion vehicles. Although performance declined (e.g., MTST dropped from 73% to 50% on single segments), time-shift aggregation restored the accuracy to acceptable levels.

The work in [81] presented a driver profiling framework that analyzed temporal driving behavior using OBD-II time-series data. To investigate feature redundancy in high-dimensional sensor data, the authors implemented a customized Maximum Relevance Minimum Redundancy (mRMR) feature selection technique. This approach is rooted in conditional likelihood maximization and was used to filter 51 raw vehicle parameters from the public OBD-II dataset [78] down to 15 highly relevant and minimally redundant features. The selected attributes exhibited discriminative power, as confirmed via statistical analysis using box plots and correlation heatmaps. The dataset comprised vehicle operation data from ten drivers over two path scenarios (PathOrder1 and PathOrder2). An LSTM neural network was developed to model sequential dependencies and capture intra-driver behavioral consistency. The model architecture included three hidden LSTM layers and a Softmax output layer. It was trained using the Adam optimizer and evaluated using sparse categorical cross-entropy loss. Training/testing was performed with an 80/20 split and 5-fold cross-validation to ensure generalizability. The LSTM model achieved an overall test accuracy of 94% on the combined dataset, with 98.4% and 96.6% accuracy for PathOrder1 and PathOrder2, respectively. This work concluded that temporal modeling combined with strategic feature selection can support driver identification using OBD-II data.

The work in [82] addressed driver identification using supervised learning applied to OBD-II-derived driving behavior data. The underlying assumption was that each driver exhibits a unique driving style characterized by acceleration, speed, and braking patterns. The study used the KIA/Ocslab OBD-II dataset, which comprises data from ten drivers over similar 23-kilometer trips and includes 51 features. Standard preprocessing steps such as normalization and sliding-window segmentation were performed. In addition, feature selection was employed to remove homogeneous, irrelevant, or highly correlated attributes. Fifteen key features were retained, including long-term fuel trim, intake air pressure, accelerator pedal value, fuel consumption, engine torque, transmission oil temperature, and wheel speeds. The classification algorithms employed were naïve Bayes, LR, kNN, REP Tree, and SVM, while the evaluation metrics included accuracy, precision, recall, and F1-score. The results showed high classification accuracy with a small number of drivers (up to 100% accuracy with two drivers using kNN); however, the accuracy decreased significantly with an increased number of drivers (74% for ten drivers).

In [83], the authors proposed an LSTM-based driver identification framework leveraging OBD-II data to classify drivers based on individual driving patterns. Their DL-based architecture employed LSTM networks for handling sequential temporal data. These LSTMs exploited and maintained long-range dependencies via internal cell state vectors and gated memory mechanisms. A total of 54 features retrieved from a public OBD-II dataset [78] were initially captured, of which 53 were retained after preprocessing (feature selection, outlier handling via robust scaling, and deduplication). Model training used a five-fold cross-validation strategy to ensure generalization and robustness. The LSTM model outperformed classical ML methods (SVM, kNN, DT, and MLP) across all key evaluation metrics, achieving over 99% accuracy along with high precision, recall, and F1-score, which significantly outperformed DT (98.4% accuracy) and MLP (97.5%). It should be noted that the proposed model is in principle compatible with in-vehicle systems such as Automotive Grade Linux (AGL) and Android Auto in real-time embedded environments.

**Table 8:** Overview of ML-based approaches for driver identification.

Reference	Objective	ML Models	Driving Environment	Type of Vehicle	OBD or OBD-Derived Parameters	Performance Metrics
Govers et al., 2024 [80]	Driver identification	Bidirectional LSTM with Attention (BiLSTM-A) and Modified Time Series Transformer (MTST)	Korean dataset	Korean vehicles	Propulsion-system-independent features (14 features)	Accuracy in driver classification based on monitored duration.
Singh et al., 2024 [81]	Driving behaviour capturing and profiling with the objective to identify	LSTM for behaviour evolution	Korean dataset	Korean vehicles	15 OBD-II features selected based on Maximum Relevance Minimum Redundancy	Accuracy in driver identification.
Khan et al., 2023 [82]	Driver identification	Naive Bayes, LR, kNN, REP Tree, and SVM	Korean dataset, 10 drivers in similar trips (covering 23 km)	Korean vehicles	15 features (out of 51) including fuel trim, air pressure	Accuracy of driver identification.
Manderna et al., 2022 [83]	Driver identification	LSTM	Korean dataset, 10 drivers in similar trips (covering 23 km)	Korean vehicles	53 OBD-II features have been employed after preprocessing	Accuracy, precision, recall, and F1-score, compared with kNN, SVM, DTs, MLP.

## 6. Anomaly Detection and Cybersecurity Issues

Ensuring vehicle reliability and security is crucial in modern transportation, with real-time diagnostics and anomaly detection preventing failures. However, anomaly detection is often absent in traditional systems or is based on simplistic thresholds that lack adaptability. On the other hand, cybersecurity concerns related to OBD-II interfaces have intensified with the rise of connected vehicles, highlighting the need for robust IDS and protective measures. Traditional OBD-II systems lack sufficient security and rely on basic or no authentication, making them vulnerable to attacks such as message injection. Recent research has demonstrated that combining OBD-II data and ML can facilitate predictive maintenance, early fault detection, and prompt responses to cybersecurity threats. Within this framework, the current section surveys recent ML-based techniques for anomaly detection and in-vehicle cybersecurity, as summarized in Table 9.

**Table 9:** Overview of ML-based approaches for anomaly detection and protection against cybersecurity threats.

Reference	Objective	ML Models	Driving Environment	Type of Vehicular Data Issues and Threats	OBD or OBD-Derived Parameters	Performance Metrics
Andrade et al., 2024 [84]	Detection and correction of outliers in real-time OBD-II data streams on resource-constrained edge devices	TEDA and RLS	Speed variations, particularly in areas with speed bumps where speed reductions are required	Presence of outliers in vehicle sensor data that can impact monitoring, predictions, and decision-making	Speed data	Accuracy, F1 score, and recall for outlier detection, RMSE and MAE for outlier correction performance.
Dini et al., 2023 [85]	Real-time detection of cyber-attacks on the CAN bus using ML-based ECU fingerprinting	ANNs	Simulated CAN network environment	Unauthorized access, replay attacks. DoS, spoofing attacks, physical layer attacks	CAN bus voltage signal fingerprinting	Accuracy of anomaly detection, classification performance of known vs. unknown ECUs, robustness to temperature variations.
Malik et al., 2023 [86]	Anomaly detection in ransomware propagation	kNN	Simulated environments, particularly ride-hailing services with EVs moving through urban areas	Ransomware threats in connected vehicles, including hotspot-based malware infections, OBD dongle-based infections, and malicious OTA updates	Vehicle speed, vehicle acceleration/deceleration	Infections per minute, system efficiency (ratio of completed trips to total trips), financial impact (loss in earnings per hour), update convergence (percentage of EVs receiving software updates).
Aloqaily et al., 2025 [87]	In-vehicle communication security through an IDS specifically designed for the CAN-bus, focusing on detecting unusual patterns	DTs, RF, Naïve Bayes, LR, XGBoost, LightGBM, and MLP	Connected and autonomous vehicles operating in real-time environments	DoS, fuzzy attacks, RPM spoofing, gear spoofing, replay and impersonation attacks	CAN-bus data	Accuracy, precision, recall, F1-Score, FPR, and FNR.
El-Gayar et al., 2024 [88]	Intrusion detection in IoV systems, addressing vulnerabilities to cyber-attacks with high accuracy and low false negatives	RF, ET, LightGBM, and XGBoost	Connected and autonomous vehicles within the IoV ecosystem, with a focus on both intra-vehicle and inter-vehicle communication	DoS, DDoS, fuzzy attacks, spoofing (gear and gauge), false data dissemination, and sybil attacks	CAN traffic, including features such as timestamp, CAN ID, DLC data bytes, and CAN packet	Accuracy, precision, recall, F1-Score, and execution Time.

In [84], TEDA-RLS emerged as a TinyML solution for real-time outlier detection and correction in vehicular data using an OBD-II scanner. By addressing anomalies that compromise diagnostics and predictive maintenance, it overcomes traditional ML limitations such as large datasets, high computation, and centralized processing by enabling incremental learning on resource-constrained edge devices, thereby reducing cloud reliance while maintaining data accuracy. The proposed TEDA-RLS algorithm integrates two fundamental techniques: TEDA for detecting outliers in streaming data, and Recursive Least Squares (RLS) filtering for adjusting detected anomalies while ensuring data consistency. The algorithm was evaluated using both simulated and real-world datasets. Simulated power measurements from an energy substation with injected outliers enabled controlled testing. The real-world dataset comprised vehicle speed and other OBD-II parameters collected from a passenger car using a Freematics ONE+ OBD-II scanner (Freematics, Sydney, Australia). Data were processed in real-time on the scanner’s embedded ESP32 microcontroller, enabling TEDA-RLS execution without external computing. Experimental results revealed TEDA-RLS’s high accuracy in detecting and correcting outliers, achieving a low MAE of 22.23 and RMSE of 124.39 on the simulated power dataset. With real-world vehicular data, TEDA-RLS was able to detect speed anomalies during low-speed conditions, including speed bumps, traffic congestion, and sudden braking. Processing times were efficient, with TEDA at 74.25 μs, RLS filtering at 169.38 μs, and total execution at 243.63 μs per cycle, which is suitable for real-time smart vehicle deployment.

With growing vehicular network interconnectivity, cyber threats exploiting unsecured CAN bus protocols have surged, enabling data interception, function manipulation, and denial-of-service (DoS) attacks. To counteract these risks, the work in [85] proposed a real-time anomaly detection system to secure CAN bus communications, focusing on vulnerabilities introduced by remote access points (i.e., Wi-Fi, Bluetooth, and cellular networks) and OBD-II interfaces in modern vehicles. The proposed system combined ECU fingerprinting with an ANN-based IDS for CAN traffic monitoring, offering a lightweight and cost-effective alternative to cryptographic or statistical methods. The approach involved three stages: data acquisition and ECU fingerprinting, ANN-based anomaly detection, and experimental validation using CAN attack simulations and robustness tests. Data acquisition used ECU fingerprinting by extracting voltage signal characteristics from CAN bus messages. Each ECU exhibited a unique electrical signature captured by an NXP S32K144 microcontroller (NXP Semiconductors, Eindhoven, Netherlands) acting as a Traffic Analyzer with high-speed analog-to-digital converter (ADC) sampling. Features such as amplitude, skewness, kurtosis, and standard deviation were used to train a feed-forward neural network in TensorFlow Lite for efficient embedded execution. Evaluation included three CAN cyberattacks (replay, impersonation, and message injection) on a testbed with five ECUs and two attacker devices. The system achieved high detection accuracy across attacks, outperforming rule-based IDS by detecting anomalies without prior attack knowledge. Additionally, robustness tests under 24–83 °C conditions showed classification accuracy above 98.4%, confirming resilience to environmental variations and reliable protection in real-world conditions.

The work in [86] studied the business impact of ransomware on connected vehicles, shedding light on the growing cybersecurity risks in modern vehicle networks. As connected vehicles increasingly depend on remote access technologies and OBD-II interfaces, they face growing risks from ransomware attacks. Unlike traditional IT ransomware, vehicular ransomware threatens transportation systems, autonomy, and ride-hailing services. This work modeled three infection vectors: (1) hotspot attacks via compromised Wi-Fi and fog-based communications; (2) OBD dongle attacks spreading through Universal Serial Bus (USB) or infotainment and vehicle-to-vehicle (V2V) networks; and (3) malicious over-the-air (OTA) updates. It also assessed ransomware’s economic impact on ride-hailing by using a fog computing architecture to analyze propagation, enable rapid response, and quantify the financial impact of ransomware on a ride-hailing fleet by tracking earnings per vehicle per hour before and after infection. Simulations showed ransomware attacks could reduce fleet earnings by up to 45% per hour due to downtime and service loss. A two-stage mitigation using a kNN classifier to detect anomalies in CPU, memory, and network activity reduced losses to 26%, proving that early detection is effective. Experiments revealed that hotspot and OTA attacks caused moderate infection; on the other hand, OBD-II dongle attacks had the highest impact, potentially disabling vehicles. Additionally, the framework improved OTA update rates from 56% to 68% under ransomware conditions.

In [87], the authors presented a supervised ML framework for real-time intrusion detection in connected and autonomous vehicles (CAVs). They developed an IDS for the CAN bus that utilizes ML to detect malicious activity within vehicle networks. The proposed framework leveraged real-world automotive network datasets containing various cyberattacks, including spoofing, DoS, and fuzzy attack types. Seven supervised ML algorithms were applied to classify normal and malicious CAN messages: DTs, RF, naïve Bayes, LR, XGBoost, LightGBM, and MLP. The dataset used for training and evaluation was gathered from real vehicle data via the OBD-II port, capturing CAN bus traffic both during normal operations and under cyberattack scenarios. The preprocessing phase involved feature selection, normalization, and hexadecimal-to-decimal conversion to enhance model efficiency. In this work, binary classification (normal vs. attack) and multi-class classification (distinguishing between attack types) were compared to assess the models’ effectiveness in intrusion detection. Performance evaluation was based on metrics such as accuracy, precision, recall, F1-score, false positive rate (FPR), and false negative rate (FNR). Experimental results showed that the IDS framework achieved near-perfect accuracy, with RF and LightGBM reaching 99.9%. RF performed best on merged datasets, balancing accuracy and low computational cost, while LightGBM excelled in binary classification with similar efficiency. DT offered the fastest execution, making it ideal for real-time deployment, whereas MLP had high computational demands, limiting its real-time usability despite strong multi-class performance. This study emphasized the need for lightweight models, with edge deployment reducing latency and resource usage to enable practical in-vehicle cybersecurity.

In [88], a Smart Collaborative IDS (CIDS) was proposed to secure vehicular networks in Internet of Vehicles (IoV) environments using an ensemble ML model. The openness and complexity of IoV networks make them highly vulnerable to cyberattacks such as DoS, sybil, and false data injection attacks, all of which can undermine safety and operational efficiency. To mitigate these risks, the authors introduced the Dynamic Forest-Structured Ensemble Network (DFSENet), an advanced IDS designed to enhance detection accuracy, reduce false negatives, and improve scalability in IoV environments. The DFSENet framework incorporates data-balancing techniques such as the Synthetic Minority Over-sampling Technique (SMOTE) and PCA to address class imbalance and optimize feature dimensionality. Specifically, this work used two benchmark datasets for evaluation: the CICIDS2017 dataset for general network intrusion detection and the Car-Hacking dataset for automotive cybersecurity threats. Furthermore, the intrusion detection model integrated multiple ML classifiers, including RF, Extra Trees (ET), LightGBM, and XGBoost, which were structured within a multi-layered ensemble network to improve accuracy and generalizability. The DFSENet architecture supports dynamic-depth learning, which automatically adjusts the network complexity based on validation performance to optimize both detection efficiency and computational overhead. Experimental results showed that DFSENet significantly outperforms conventional ML models, achieving an overall accuracy of 99.2% on the CICIDS dataset and 98% on the Car-Hacking dataset, with high precision (95.6%), recall (98.8%), and F1-score (96.9%). A comparative analysis also revealed that DFSENet surpasses conventional models such as DTs, MLP, and CNNs, particularly in detecting sophisticated attack patterns.

## 7. Intelligent Road Perception and Driving Support

While conventional OBD-II systems provide raw data for basic diagnostics, they lack the processing power to support advanced applications such as ADAS or ITS; for instance, they cannot classify road surfaces or navigate in GPS-denied environments without external systems. The use of advanced ML techniques is poised to revolutionize road perception, vehicle navigation, and driving support. By harnessing real-time vehicle data, these techniques enable cost-effective road quality assessment, accurate trajectory prediction during GPS outages, and precise velocity correction for state estimation in Satellite Navigation (SatNav)-deprived environments. This section explores how the ML-based solutions outlined in Table 10 contribute to this domain.

In [89], OBD-II, accelerometers and GPS devices were used to classify road surfaces according to the Pavement Surface Evaluation and Rating (PASER) system [90], a standard used to identify road segments in need of repairs or reconstruction. Road conditions were assigned scores from 1 to 10, with lower scores indicating deteriorated road segments requiring maintenance or reconstruction. These ratings were grouped into three broad categories: “Good” (6–10), “Fair” (4–5), and “Poor” (1–3). The data collection process involved two vehicles equipped with Gen 2.5 Danlaw DL860 OBD-II data loggers (Danlaw, Inc., Novi, MI, USA), which collected accelerometer readings. A total of 4935 s of “Good” road data, 36,329 s of “Fair” road data, and 13,583 s of “Poor” road data were collected. Preprocessing included GPS-based geofencing to match the data with PASER-labeled road segments. Feature extraction involved statistical measures such as the mean, standard deviation, root mean square (RMS), and range for each accelerometer axis (X,Y,Z), along with Fast Fourier Transform (FFT) features for frequency domain analysis. Four ML models for road classification were evaluated: Ordinal LR, SVM, ANN, and CNN. The ordinal regression model achieved 67.3% accuracy but struggled with “Poor” (18.1%) and “Good” (1.2%) roads. The SVM model performed slightly worse at 53.9%, classifying 37.9% of "Poor" roads and 1.1% of "Good" roads, which dropped to 49.5% when trained on raw data. The ANN model showed better results, with 68.6% accuracy on engineered features, identifying 33.8% of “Poor” roads and 6.7% of “Good” roads. When trained on raw data, this improved to 44.8% and 18.2%, respectively. The CNN extracted features automatically, achieving 65.6% accuracy overall, and excelled at classifying “Poor” (53.5%) and “Good” (27.5%) roads. Despite some overfitting (98.9% training accuracy), the CNN outperformed the traditional models in detecting which road segments required maintenance.

In [91], a trajectory data acquisition framework was presented to enhance vehicle tracking in urban environments. The proposed approach was intended to address challenges in GPS-denied areas where signal blockages affect positioning reliability, for which a cost-effective GPS module and OBD reader were used to correct GPS errors with motion sensor data. The proposed system enabled continuous vehicle positioning without requiring additional Inertial Measurement Unit (IMU) devices, making it a scalable solution for private car trajectory monitoring. Moreover, an ensemble learning-based Gaussian Process Regression (GPR) method was employed to model trajectory data, tackling nonlinearity, nonstationarity, and incremental learning challenges. To enhance adaptability, a regression-to-classification (R2C) approach was implemented and integrated with Learn++, enabling incremental learning when new trajectory data were introduced. This method dynamically adjusts to evolving driving conditions and resolves concept drift problems in trajectory prediction. The experimental setup involved installing a GPS/OBD integration device in a 2016 Ford Edge SUV to collect motion sensor data, including speed, steering direction, and RPM. Trajectory data were transmitted via LTE to a server for analysis. Field tests were conducted under various road conditions, with simulated GPS outages of 30–45 s. The GPR-based model outperformed baseline methods, achieving an RMSE of 10 m on sharp turns and 6 m on viaducts, while alternative methods exceeded 33 m. This model closely matched the true trajectories in straight road and highway scenarios even during GPS outages up to 45 s. On complex roads, such as left-angle turns and viaducts, RMSE remained as low as 6–10 m, significantly outperforming competing models that exceeded 33 m.

In [92], an ML-based approach was proposed to generate correction data for improving vehicle state estimation in autonomous driving and ADAS. This work addressed the challenge of degraded position, velocity, and Course Over Ground (COG) accuracy in environments with poor SatNav signals, such as tunnels, urban canyons, or parking structures. Traditional methods rely on Inertial Navigation Systems (INS), which integrate inertial sensors with SatNav correction data; however, their accuracy diminishes over time in SatNav-deprived environments. To tackle this issue, the authors introduced a Transformer Neural Network (TNN) model that utilizes OBD-II data to generate accurate velocity correction data, thereby enhancing INS performance and ensuring precise vehicle state estimation even in the absence of SatNav signals. In this context, a test vehicle equipped with high-precision reference sensors (Automotive Dynamic Motion Analyzer (ADMA, GeneSys Elektronik GmbH, Offenburg, Germany) and Correvit S-Motion (Kistler Instrumente AG, Winterthur, Switzerland)) was used to generate a dataset containing 3,428,099 OBD measurements and 7,691,147 reference sensor measurements, covering approximately 535 km of driving distance. Moreover, the dataset was processed to synchronize OBD readings with reference sensor outputs to ensure data reliability. Experimental results demonstrate the effectiveness of the TNN-based correction model. Using the full dataset, the model achieves an MAE of 0.167 km/h (0.046 m/s) and RMSE of 0.240 km/h, closely matching the accuracy of high-end reference sensors. This work also explored the impact of dataset size on model performance, revealing that reducing the training data to 5000 samples increased the MAE to 0.863 km/h (0.24 m/s), which highlights the importance of large datasets for robust velocity estimation.

**Table 10:** Overview of ML-based approaches for intelligent road perception and driving support.

Reference	Objective	ML Models	Driving Environment and Type of Road	OBD or OBD-Derived Parameters	Performance Metrics
Sabapathy et al., 2023 [89]	Low-cost solution for road surface classification using standardized OBD-II accelerometer data	CNN, ordinal LR, SVM, and ANN	Naturalistic driving conditions, Asphalt roads, classified into three categories: ‘Good’, ‘Fair’, and ‘Poor’.	3-axis accelerometer data (X, Y, Z axes), vehicle speed, GPS data	Overall classification accuracy, accuracy per road class (Poor, Fair, Good)
Xiao et al., 2022 [91]	Low-cost and user-friendly method for large-scale car trajectory data acquisition in urban environments, especially during GPS outages	GPR, R2C with Learn++	Real-world urban environments with complex conditions like straight roads, highways, turns, and viaducts	Velocity, steering direction, RPM, GPS position (latitude, longitude)	RMSE, AE, accuracy of predicted trajectories
Flores Fernández et al., 2023 [92]	Highly accurate velocity correction data, ensuring accurate vehicle state estimation for autonomous driving and ADAS in environments with limited SatNav coverage	TNN	Test track, primarily asphalt roads, simulating real-world driving conditions, with varied speeds (0–50 km/h) and diverse driving patterns	Vehicle velocity, individual wheel velocities, steering angle	MAE, RMSE, and inference time

## 8. Lessons Learned and Research Insights

This section distills key findings from the reviewed works, outlining the current state and potential of ML-driven OBD-II systems.

- **Fuel and Energy Monitoring, Estimation, and Optimization**
  - *Feature Importance:* Multiple studies identify OBD-II parameters such as engine load, vehicle speed, coolant temperature, and short-/long-term fuel trims as strong predictors for accurate fuel and energy estimation.
  - *Model Selection Tradeoffs:* RF algorithms consistently outperform traditional ANNs in real-world HDDV datasets due to better accuracy and interpretability. However, LSTM networks provide superior performance in dynamic environments where time dependencies are significant. The tradeoff lies in LSTMs’ higher computational cost and data requirements, making RF more suitable for real-time and resource-constrained settings.
  - *Behavioral and Environmental Generalization:* Robustness to diverse driving behaviors and environmental factors (e.g., road topology, traffic, weather) remains a key challenge. Urban driving introduces high variance, which limits model transferability. Methods such as domain adaptation, transfer learning, and data augmentation are underutilized but promising.
  - *Vehicle-Specific Characteristics:* In heavy-duty vehicles, propulsion energy is heavily influenced by real-time weight/load variations. Incorporating vehicle mass into models improves precision, while neglecting it—especially during frequent load/unload transitions—leads to substantial estimation errors.
  - *Edge-Based Energy Feedback:* ML models deployed on embedded or smartphone-based edge devices provide real-time energy feedback without cloud dependency. Benefits include reduced latency, enhanced data privacy, and lower communication costs, facilitating personalized eco-driving systems via platforms such as TensorFlow Lite.
  - *Infrastructure-Aware Estimation:* Including road infrastructure features (e.g., traffic signals, intersections, elevation) significantly enhances model accuracy. LSTM-based models are especially effective at capturing these context-driven patterns, particularly in mixed urban–rural routes.
  - *Scalable Deployment:* The low cost and plug-and-play nature of OBD-II ports support scalable eco-driving deployments across personal and fleet vehicles when paired with Bluetooth and mobile/cloud apps. While cloud synchronization is common, local computation offers faster and more privacy-preserving solutions.
- **Emission Control and Environmental Impact**
  - *Model Performance and Generalizability:* ML models trained on large OBD-II datasets enable accurate real-time estimation of emissions such as NO_*x*_, CO_2_, and particulate matter. However, generalizability across vehicle types, conditions, and regions remains a challenge due to dataset and sensor variability.
  - *NO_x_ Emissions Insights:* NO_*x*_ emissions are primarily driven by high-temperature combustion, engine load, gradient, and SCR efficiency. While EGR reduces NO_*x*_, it may increase particulate emissions and degrade downstream components such as DPFs. ML-based monitoring (e.g., RF, Seq2Seq) outperforms threshold-based OBD-II methods in detecting SCR faults and predicting NO_*x*_ spikes.
  - *Soft Sensor Integration:* Virtual sensors built using ML provide a cost-effective alternative to physical emission sensors, enabling closed-loop control of SCR and DPF systems without the need for extensive lab calibration.
  - *CO_2_ Emissions and Fuel Type:* Flexible-fuel hybrid vehicles using ethanol emit less CO_2_ than gasoline counterparts. ML models can optimize fuel-switching strategies to maximize environmental benefits based on driving conditions.
  - *Black Carbon and Load Dependence:* BC emissions from gasoline direct-injection engines rise nonlinearly with engine speed and load. Effective mitigation requires a combination of combustion system design and post-treatment (e.g., gasoline particulate filters).
  - *Regulatory Compliance:* ML-based emission estimators outperform traditional tools such as COPERT, supporting compliance with modern emission standards through higher estimation accuracy.
- **Driving Behavior and Driver Analysis**
  - *Behavioral Feature Extraction:* Raw OBD-II signals (e.g., RPM, throttle position, acceleration) can be transformed into high-level driving events (e.g., hard braking, sharp turning) via heuristics or supervised ML, enabling profiling across a behavioral spectrum from conservative to aggressive.
  - *Contextual Data Fusion:* Integrating contextual factors (e.g., time of day, road type, and weather) improves behavior classification. For instance, braking on a highway carries different implications than in city traffic. Context-aware classifiers are better able to handle such nuance.
  - *Safety Feedback Loops:* ML models can detect unsafe driving patterns in real time and provide feedback to drivers. Certain systems gamify performance metrics to encourage safer and more efficient driving habits.
  - *Driver Identification and Authentication:* RNNs and LSTM models can identify drivers by using micro-patterns in throttle/brake usage and gear shifts. While promising, intra-driver variability makes this task harder than simple behavior classification.
  - *Vehicle Dependency:* Driver analysis models are sensitive to vehicle-specific attributes such as powertrain type, fuel system, and model year. Many studies overlook these factors, potentially affecting cross-vehicle generalization.
  - *Data Scarcity and Quality:* Most commercial OBD-II devices have low sampling rates, and many studies rely on small datasets; thus, building large, high-resolution, and vehicle-specific datasets is critical for improving model robustness.
  - *Data Integrity and Security:* As OBD-II-based behavior profiling gains adoption in insurance and legal domains, data authenticity becomes crucial. Blockchain-based methods offer promising solutions to prevent tampering and spoofing.
- **Vehicle Health Monitoring, Anomaly Detection, and Cybersecurity**
  - *Anomaly Detection in Real Time:* Lightweight techniques (e.g., autoencoders, clustering, and statistical profiling) are commonly used to detect real-time anomalies in OBD-II signals, ensuring timely alerts without overwhelming computation.
  - *Edge Deployment of Security Models:* Implementing anomaly/intrusion detection directly on embedded platforms reduces latency, preserves bandwidth, and ensures localized response, all of which are vital for safety-critical automotive systems.
  - *Cyberattack Scenarios and Resilience:* Simulated attack scenarios (e.g., false injection, message delay, spoofing) demonstrate that ML classifiers trained on time series data can differentiate between legitimate and malicious activity. Evaluation setups with embedded microcontrollers (e.g., NXP S32K144) validate feasibility, with detection accuracy exceeding 98% across temperature ranges (24–83 °C).
- **Intelligent Road Perception and Driving Support**
  - *OBD-II and ML for Road Monitoring and Vehicle Trajectory:* ML models using OBD-II data (e.g., accelerometer, speed) can classify road conditions and support trajectory estimation, which is especially useful in GPS-denied environments when combined with inertial sensors.
  - *Challenges in Data Handling and Model Choice:* Accurate road classification requires careful preprocessing such as geofencing and sensor normalization. Traditional ML (e.g., SVM) struggles with these tasks, while DL models (e.g., CNNs) perform better; however, these models require mitigation techniques to prevent overfitting and ensure generalizability.
  - *ADAS Enhancement in GPS-Limited Areas:* Integrating OBD-II with other vehicle sensors improves ADAS features such as collision avoidance and lane keeping by ensuring reliable state estimation even when GPS signals are lost.
  - *Scalability and Real-World Validation:* Field tests confirm OBD-II’s practicality for large-scale road monitoring. Large and diverse datasets are essential for ensuring ML model performance across varied conditions.
  - *Dynamic Adaptation to Evolving Conditions:* Incremental learning allows ML models to adapt to changes in road conditions and driving styles over time, supporting robust real-time performance in dynamic environments.

It is also worth noting that although low-cost OBD-II scanners make data collection technically feasible, many studies rely on pre-existing datasets, typically multivariate time-series recordings from real-world or controlled driving sessions. These datasets are often labeled by driver, vehicle, scenario, or event and provided in CSV or JSON formats, with durations ranging from minutes to hours. High-quality datasets feature diverse drivers, routes, and behaviors along with rich sensor coverage, comprehensive metadata, and balanced distributions. The sampling frequency (commonly 1 Hz or higher) depends on the type of scanner, with professional-grade tools enabling sub-second intervals.

### Effectiveness of ML Models

ML has become integral to enhancing the capabilities of OBD-II-based vehicle intelligence systems. However, the effectiveness of these models depends strongly on the specific operational context, sensor availability, data modality, and computational constraints. This subsection presents a synthesis of the most successful ML approaches organized by both model type and the environment in which they perform best.

- **Sequential DL Models (LSTM, GRU, Transformers):** These models are especially effective in environments characterized by temporal variability and high-frequency sensor data. LSTM and GRU architectures outperform classical models in tasks such as fuel consumption estimation and driver behavior profiling in *urban traffic and variable terrain*, where stop-and-go dynamics and frequent elevation changes are prevalent. Hybrid LSTM-Conv and attention-augmented models (e.g., GRU + attention) further improve accuracy by focusing on key time segments and contextual dependencies. Transformers, with their long-range temporal modeling capability, are best suited for *SatNav-denied conditions* such as tunnels or urban canyons, where they enable precise vehicle velocity corrections using inertial and OBD-derived data.
- **Hybrid and Feature Fusion Architectures:** Models that combine sequence learning with feature selection or optimization, such as PA-LSTM or LSTM with PSO, have shown strong performance in *multi-pollutant emission prediction*. These architectures effectively handle heterogeneous input streams such as SCR temperature, fuel rate, and load conditions, resulting in enhanced prediction accuracy under different driving patterns.
- **Ensemble Methods (RF, Gradient Boosting, SVR):** Ensemble models excel in *highway and structured driving* environments where driving behavior is smoother and less stochastic. RF and SVR provide accurate estimations for fuel efficiency, emission compliance, and anomaly detection due to their robustness and interpretability. Moreover, in *post-crash and safety profiling*, classifiers such as LightGBM and RF are frequently employed to classify crash severity and abnormal driving behaviors from OBD logs and sensor snapshots, offering explainable and efficient inference.
- **CNNs and GPR-Based Ensembles:** CNNs are well suited for *road surface classification* tasks, particularly when raw sensor signals (e.g., accelerometers or vibration data) are available. They offer automatic hierarchical feature extraction, which improves detection of poorly maintained road segments. In trajectory prediction under *GPS signal loss*, ensemble models built on GPR with incremental learning capabilities provide strong adaptability to changing vehicle dynamics and can outperform traditional dead reckoning methods.
- **Lightweight and Incremental Models (TEDA-RLS, kNN, SVM, ANNs):** These models are ideal for *resource-constrained deployments* such as low-power ECUs or embedded edge devices. TEDA-RLS supports real-time anomaly correction with minimal latency and memory usage, making it suitable for in-vehicle diagnostics and early fault detection. Similarly, shallow ANNs, SVMs, and kNN classifiers are used in scenarios such as outlier detection and cybersecurity threat identification (e.g., ransomware detection via CPU/memory patterns), offering acceptable accuracy without sacrificing responsiveness.

**Figure 5:**
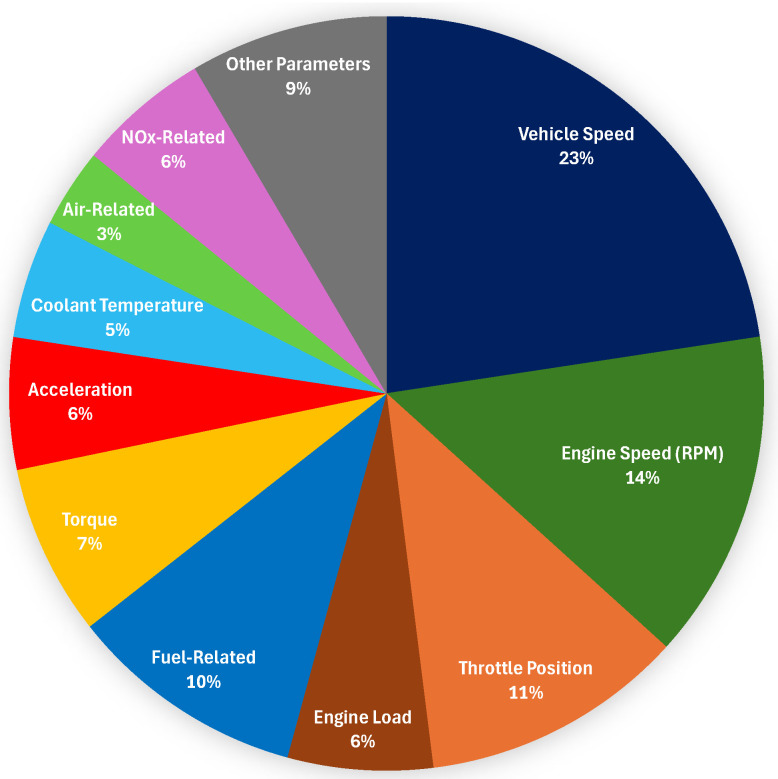
Distribution of feature categories used in ML-based models for vehicular data analysis.

Table 11 shows a summary of suggested ML-based methods for optimizing vehicle management across various optimization problems. Each method has been selected based on performance metrics such as RMSE, MAE, MAPE, R^2^, and accuracy and tailored to specific contexts, including heavy-duty trucks, light vehicles, or electric vehicles. Figure 5 illustrates the frequency of feature categories used in ML-based models for vehicular data analysis. Vehicle speed and engine speed (RPM) appear most frequently, reflecting their critical role in monitoring vehicle dynamics. Throttle position, fuel-related parameters, and torque are also commonly utilized, supporting models focused on engine efficiency and driver behavior. Less frequent but still significant features such as NO_*x*_-related metrics and air intake parameters contribute to emission monitoring and environmental impact assessments.

**Table 11:** Summary of suggested ML-based methods for vehicle management optimization problems.

Optimization Target	Suggested Method	Key Performance Metrics
Fuel Consumption (HDTs)	LSTM-Conv	MAPE: 9.81% (FCR), 1.49% (trip fuel economy)
Fuel Consumption (Light Vehicles)	Elman Neural Network	RMSE: 3.672 L/km, γ: 98.27%
SOC/RDR for EVs	Nonlinear SVR with RBF Kernel	R^2^: 0.95, MAE: 2.4%
NO_*x*_ Emissions	Seq2Seq neural network (Bi-GRU with Attention, ITL)	RMSE reduction: 62% overall, 73.6% high-emission
CO_2_ Emissions	XGBoost	R^2^: 0.942 (HDDVs), 0.981 (UHDVs)
Multi-Pollutant Emissions	PA-LSTM	Strong RMSE, MAE, MAPE, R^2^
Safety Monitoring	J48 DT	Accuracy: 100%, Precision: 100%, Recall: 100%
Driving Behavior Profiling	RF	Accuracy: 100%
Driver Identification	LSTM	Accuracy: >99%, high precision/recall/F1
Anomaly Detection	TEDA-RLS	MAE: 22.23, RMSE: 124.39, 243.63 μs/cycle
Cybersecurity	RF, LightGBM	Accuracy: 99.9%
Road Surface Classification	CNN	Accuracy: 65.6%, Poor: 53.5%, Good: 27.5%
Trajectory Prediction	GPR with R2C and Learn++	RMSE: 6–10 m
Velocity Correction	TNN	MAE: 0.167 km/h, RMSE: 0.240 km/h

## 9. Challenges, Gaps, and Future Research Directions

Although ML is reshaping OBD-II systems, real-world deployment depends on addressing significant technical, practical, and regulatory hurdles. This section identifies current challenges and research gaps followed by a discussion of strategic directions for developing scalable, intelligent, and privacy-aware automotive systems.

### 9.1. Challenges and Gaps in ML-Enabled OBD-II Systems

Below, the challenges and research gaps in the field are organized into five thematic domains, encompassing both algorithmic/data issues and practical implementation concerns.

- **Data Quality, Preprocessing, and Generalization**
  - *Sensor degradation, noise, and latency:* OBD-II signals are often affected by measurement errors, especially under harsh conditions; for example, NO_*x*_ sensors have shown over 40% error rates in some studies. These signal imperfections reduce model reliability and predictive performance.
  - *Biased and limited datasets:* Many models are trained on narrowly scoped datasets that do not reflect the variability in vehicle types, road conditions, or climate zones. This limits generalization to diverse operational settings, highlighting the need for broader and more heterogeneous datasets.
  - *Need for robust preprocessing:* Advanced preprocessing techniques such as denoising, interpolation, and cross-sensor validation are essential for cleaning OBD-II inputs prior to learning. These must also be coupled with domain-specific feature engineering and large-scale dataset curation covering heterogeneous platforms.
- **Scalability, Efficiency, and Model Optimization**
  - *Embedded hardware limitations:* Deep models such as MLPs, CNNs, and LSTMs often exceed the processing and memory capabilities of in-vehicle ECUs. Lightweight alternatives such as RF, LightGBM, and DFSENet are more suitable for real-time edge deployment. Edge-aware pruning, quantization, and distillation techniques are also promising for deployment in constrained environments.
  - *Model complexity vs. accuracy tradeoffs:* Achieving high accuracy while maintaining computational efficiency remains a core challenge, particularly for latency-sensitive applications such as anomaly detection and eco-driving feedback.
  - *Protocol fragmentation and interoperability:* Interoperability challenges primarily stem from physical and electrical variations across protocols (e.g., ISO 15765-4 CAN, older variants), especially in legacy or EVs that may lack full OBD-II support. Parameter heterogeneity due to manufacturer-specific PIDs, undocumented scaling factors, and varying byte formats limits model generalizability and functionality. Additionally, DTCs are split into standardized and proprietary forms, restricting access to advanced diagnostic insights. Operational factors such as ECU polling frequencies, protocol bandwidths, and request intervals further impact data consistency and analytics.
- **Computational Constraints, Sensor Fusion, and System Integration**
  - *Real-time inference bottlenecks:* Cloud-dependent ML models introduce latency and rely on stable connectivity, which is not guaranteed in all vehicular environments.
  - *Underutilized sensor fusion:* OBD-II data remain largely decoupled from other vehicular sensor streams. Synchronization of multi-modal data for tasks such as road condition detection or trajectory estimation remains a challenge due to bandwidth constraints and protocol incompatibility.
  - *Integration with ADAS:* Combining OBD-II analytics with ADAS poses significant challenges due to their fundamentally different architectures and requirements. OBD-II is ECU-centric, polled via a central gateway, and operates at low data rates (1–10 Hz), while ADAS is sensor-centric and its features (e.g., lane-keeping, adaptive cruise control) require high-frequency low-latency streaming from cameras, radars, and other sensors, often exceeding tens or hundreds of Hz. OBD-II primarily supports emissions and diagnostics, lacking access to dynamic vehicle data such as yaw rate, steering angle, and object detection which are crucial for ADAS. Although centralized gateways using FlexRay or Controller Area Network with Flexible Data-rate (CAN-FD) protocols [93] can facilitate low-latency fusion for enhanced situational awareness, coordination between disparate software stacks (OBD-II, ADAS, infotainment) remains a major integration barrier.
  - *Fleet-level deployment and management:* In fleet scenarios, scalability is further challenged by the heterogeneity of vehicle models, sensor aging, and driver behavior. Centralized ML model training with distributed inference, edge–cloud orchestration, and over-the-air updates require robust communication and system security protocols.
- **Cybersecurity, Privacy, and Regulatory Barriers**
  - *Expanded attack surfaces:* Diagnostic interfaces (OBD-II ports) and wireless connectivity expose vehicles to cyber threats such as spoofing, ransomware, and message injection. Current rule-based IDSs are often inadequate for detecting novel or zero-day attacks. ML-based intrusion detection and trust-aware communication layers are currently active areas of research.
  - *Data privacy and ethical compliance:* The use of behavioral and location data for model training introduces privacy risks, particularly when transmitted or processed externally. OBD-II applications capable of identifying individual drivers must comply with the EU’s General Personal Data Protection (GDPR) regulation, which requires a lawful basis for processing (e.g., consent or legitimate interest), purpose limitation, and data minimization. Real-time tracking via telemetry or cloud-based services may fall under the scope of the EU’s ePrivacy Directive [94], especially if in-vehicle communications are monitored. Emerging techniques such as Secure Multiparty Computation (SMPC) [95], differential privacy [96], and federated learning (FL) [97] offer promising privacy-preserving mechanisms.
  - *Legal barriers to cloud integration:* Transferring vehicle data to the cloud raises legal concerns over cross-border data flows, ownership, and liability for system failures, especially when third-party services are used to handle emissions or safety-critical analytics.
  - *Lack of certification pathways:* Despite high prediction performance, many ML models lack transparency, reproducibility, or explainability, which are necessary factors for legal and regulatory approval. Moreover, exposing safety-critical ADAS data over diagnostic interfaces such as OBD-II introduces security and functional safety concerns, as ADAS systems must comply with standards such as ISO 26262 [98] that require rigorous validation beyond the scope of diagnostic protocols. Standardized data formats and auditability are also needed for critical tasks such as emission verification and accident analysis.
- **Emission Monitoring, Eco-Driving, and Smart Mobility**
  - *Under-contextualized emission models:* Emission models trained on short-duration or context-poor datasets may fail to generalize across traffic types, road profiles, and ambient conditions. These models also struggle with cold-start effects and nonlinear behaviors in fuel consumption.
  - *Lack of real-time feedback systems:* Most eco-driving systems do not provide personalized context-aware feedback during vehicle operation. There is a need for ADAS-embedded ML platforms that can interpret OBD-II signals in real time and offer actionable guidance to drivers while avoiding distractions and ensuring interpretability.
  - *Smart city integration challenges:* Scaling ML models for use in fleet management or city-wide traffic optimization is hindered by high data throughput and computational overhead. Efficient representations such as trajectory compression and semantic road classification are necessary for cloud or mobile deployment. In addition, interoperability with municipal platforms and standards (e.g., DATEX II, ITS-G5 [99]) remains a challenge.

### 9.2. Future Research Directions

To address the multifaceted challenges outlined above, future research must adopt a holistic and cross-disciplinary agenda that aligns with emerging industry trends. Accordingly, the following strategic and practical directions are suggested:

- **Public OBD-II Datasets and Benchmarking:** A major barrier to reproducible and comparable research is the scarcity of publicly available, large-scale, and diverse OBD-II datasets. Future efforts must prioritize the creation and open sharing of well-annotated datasets spanning multiple vehicles, fuel types, and real-world driving conditions. Standardized benchmarks and challenge platforms inspired by industry-led initiatives such as those for connected and autonomous vehicles [100] are needed in order to evaluate anomaly detection, fault diagnosis, and predictive maintenance methods. Such platforms can facilitate collaboration between academia and industry, helping to ensure that datasets reflect real-world fleet management needs.
- **Data Quality, Preprocessing, and Generalization:** Building robust ML systems begins with high-quality and reliable data. Future work should develop comprehensive preprocessing pipelines including sensor signal denoising, time-series interpolation, and outlier rejection that are specifically tailored to the noisy and heterogeneous nature of OBD-II signals. To enhance generalization, datasets must cover broad operational domains (e.g., urban, highway, cold starts) and integrate contextual data such as weather, traffic, and digital elevation maps which are critical for industry applications such as emissions modeling. Industry adoption of edge computing for real-time data preprocessing can further improve data quality by reducing latency and enabling onboard noise filtering, aligning with the computational constraints of automotive platforms.
- **Lightweight and Efficient Model Design for Onboard Deployment:** Given the stringent computational and memory constraints of embedded automotive platforms, there is a pressing need for lightweight ML and DL models optimized for real-time onboard execution. Research should focus on model compression techniques such as pruning, quantization, and knowledge distillation as well as on the exploration of hybrid approaches combining classical ML and shallow neural networks. The industry’s shift toward edge computing necessitates models that operate efficiently on in-vehicle hardware and can support applications such as predictive maintenance and eco-driving feedback. Dynamic model adaptation techniques that scale complexity based on task demands or available resources can enable efficient and adaptive vehicle intelligence.
- **Protocol Standardization, Interoperability, and Emerging Access Methods:** Fragmentation of OBD-II implementations and proprietary protocols limits large-scale analytics and model portability. Future research should support efforts to standardize data exchange protocols (e.g., ISO 15031, SAE J1939) and develop platform-agnostic APIs and middleware that abstract protocol-specific details. This is critical for industry applications such as scalable fleet management and OTA diagnostics. Moreover, the OBD-II interface itself is evolving, particularly for EVs, where data access increasingly occurs via internal APIs or telematics platforms accessed through the Android Automotive operating system (OS), Apple CarPlay, or manufacturer-specific apps (e.g., FordPass, TeslaFi). Standardized protocols and open APIs are essential for enabling scalable cross-brand analytics and diagnostics in electrified vehicle fleets.
- **Sensor Fusion and ADAS Integration:** Enhancing predictive accuracy and situational awareness requires fusing OBD-II data with other vehicular and environmental sensor inputs. Future systems should employ structured multimodal learning approaches that can align temporally and spatially disparate data streams via synchronization protocols and centralized gateways such as FlexRay. Data fusion can improve applications such as road surface classification, autonomous navigation in GPS-denied zones, and adaptive safety interventions. In addition, industry adoption of digital twins [101] can further enhance sensor fusion and support ADAS by simulating real-time vehicle dynamics.
- **Explainable and Trustworthy AI:** As ML systems increasingly influence safety-critical decisions, their outputs must be made interpretable and auditable. Future research should incorporate explainability tools such as SHAP, LIME [102], and counterfactual reasoning in order to visualize and justify model behavior. Hybrid architectures combining rule-based logic with learned representations can enhance user trust and align with regulatory requirements. Ensuring transparency, consistency, and reproducibility of ML outputs is essential for long-term system certification, particularly for industry stakeholders such as automakers and regulators seeking auditable diagnostic systems.
- **Privacy-Preserving ML:** The deployment of ML raises legitimate concerns over the privacy of location, behavior, and biometric data. Federated learning (FL) offers a compelling paradigm in which models are trained locally on-device and only aggregated updates are shared with a central server. Future work should focus on optimizing FL architectures for vehicular environments while incorporating differential privacy mechanisms and blockchain-based audit trails to prevent tampering and unauthorized data inference. These privacy-preserving techniques are critical for industry applications, where they enable secure driver profiling and fleet analytics while ensuring compliance with data protection regulations.
- **Resilient Cybersecurity Systems:** As vehicle systems become increasingly connected, they face growing exposure to cyber threats. Future research should emphasize the development of adaptive and resilient IDS capable of learning incrementally and detecting zero-day attacks. ML techniques such as ensemble-based anomaly detection, adversarial training, and unsupervised clustering can be used to identify and mitigate novel attack patterns in real time. Combining behavioral analytics with network traffic monitoring can yield more comprehensive protection.
- **Context-Aware Emission Modeling:** Accurately estimating vehicular emissions in real-world driving conditions remains a key challenge due to non-stationary factors (e.g., cold starts, stop-and-go traffic, and elevation changes). Future models should incorporate contextual data such as digital elevation maps, ambient temperature, and traffic congestion into prediction pipelines. Advanced temporal architectures such as attention-based LSTMs and transformer models can be leveraged for more effective modeling of nonlinear and time-dependent relationships. This is particularly essential for ensuring industry compliance with evolving regulations such as Euro 7 and China-VI.
- **Scalable Eco-Driving and Smart Mobility Tools:** Future systems should integrate ML models into cloud-based or edge-deployed ADAS platforms to provide real-time eco-driving recommendations, predictive maintenance alerts, and adaptive route planning. Techniques such as trajectory compression, semantic road classification, and low-power analytics can make these solutions scalable in smart city ecosystems. Personalization based on driving behavior, vehicle type, and traffic context will be key to maximizing energy savings and user engagement.

**Figure 6:**
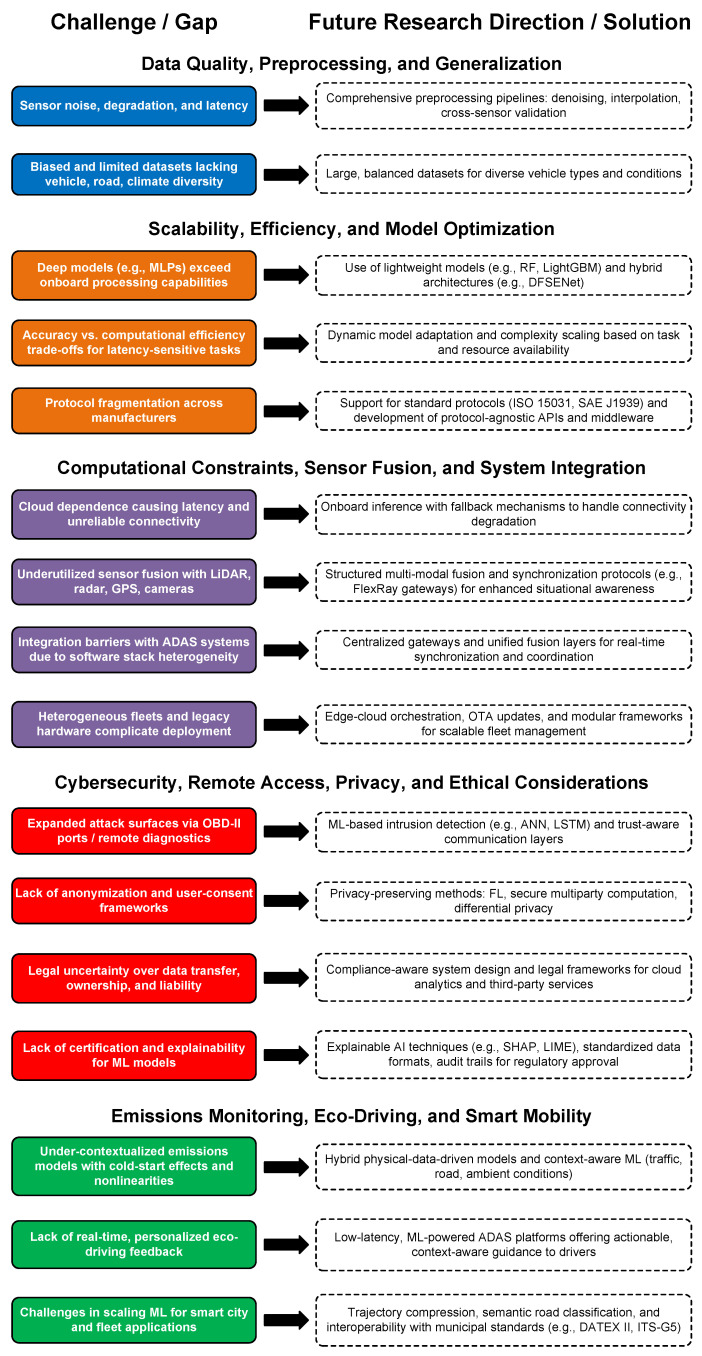
Key challenges in ML-driven OBD-II systems and corresponding potential solutions [43,45].

A structured overview of the primary challenges and gaps is presented in Figure 6 alongside corresponding future research directions and potential solutions.

## 10. Conclusions

This paper provides a comprehensive review of ML-inspired applications utilizing OBD-II data, highlighting their transformative impact across such varied automotive domains as fuel and energy optimization, emission control, driver behavior profiling, vehicle health monitoring, cybersecurity, road perception, vehicle navigation, and driving support. OBD-II parameters compose a rich set of parameters with dynamic behaviors and heterogeneous characteristics in terms of changing rates, value scaling, interpretability, and information content. Recent studies have attempted to better understand these parameters by identifying high-level driving events and behavioral patterns. The abundance of data and absence of deterministic rules make the OBD-II ecosystem particularly well suited for the application of ML models. The integration of such models has enabled the extraction of meaningful and actionable insights from raw sensor data. These models support predictive maintenance, eco-driving feedback, and intrusion detection, contributing to more sustainable, efficient, secure, and safe vehicle operation. The key findings of this paper underline the importance of high-quality datasets, proper data preprocessing, and careful model tuning in achieving scalable and generalizable results across different vehicle types and operational conditions. Despite these advancements, challenges remain around addressing data heterogeneity, limited onboard computational resources, and cybersecurity risks posed by growing vehicle connectivity. By addressing these challenges, the synergy of ML and OBD-II technologies can further advance ITS.

## Abbreviations

The following abbreviations are used in this manuscript:

ABS	Anti-lock Braking System
ADAS	Advanced Driver Assistance Systems
ADC	Analog-to-Digital Converter
AGL	Automotive-Grade Linux
AI	Artificial Intelligence
ANN	Artificial Neural Network
API	Application Programming Interface
ARIMA	Autoregressive Integrated Moving Average
ASW	Adaptive Sequence Window
BC	Black Carbon
BiGRU	Bidirectional GRU
BiLSTM	Bidirectional Long Short-Term Memory
BPSS	Brake Pedal Position Sensor
CAN	Controller Area Network
CAN-FD	Controller Area Network with Flexible Data-rate
CART	Classification and Regression Tree
CAV	Connected and Autonomous Vehicle
CBAM	Convolutional Block Attention Module
CCPS	Crankshaft and Camshaft Position Sensors
CEL	Check Engine Light
CIDS	Collaborative Intrusion Detection System
CNN	Convolutional Neural Network
COG	Course Over Ground
CSV	Comma-Separated Values
DA-RNN	Dual-Stage Attention-Based RNN
DBSCAN	Density-Based Spatial Clustering of Applications with Noise
DDD	Driver Drowsiness Detection
DT	Decision Tree
DEM	Digital Elevation Model
DFSENet	Dynamic Forest-Structured Ensemble Network
DL	Deep Learning
DLC	Data Length Code
DNN	Deep Neural Network
DOC	Diesel Oxidation Catalyst
DoS	Denial-of-Service
DPF	Diesel Particulate Filter
DPP	Dew Point Protection
DTC	Diagnostic Trouble Code
ECU	Electronic Control Unit
EGR	Exhaust Gas Recirculation
EGT	Exhaust Gas Temperature
EKF	Extended Kalman Filtering
EOP	Engine Output Power
EPB	Electronic Parking Brake
ESC	Electronic Stability Control
ET	Extra Trees
EV	Electric Vehicle
FCR	Fuel Consumption Rate
FFB	Feed-Forward Backpropagation
FFT	Fast Fourier Transform
FNR	False Negative Rate
FPR	False Positive Rate
GBDT	Gradient-Boosting Decision Tree
GBM	Gradient-Boosting Machine
GDPR	General Personal Data Protection
GMM	Gaussian Mixture Model
GPR	Gaussian Process Regression
GPS	Global Positioning System
GRU	Gated Recurrent Unit
GUI	Graphical User Interface
HC	Hydrocarbon
HDDV	Heavy-Duty Diesel Vehicle
HDT	Heavy-Duty Truck
HEV	Hybrid Electric Vehicle
IAT	Intake Air Temperature
IDS	Intrusion Detection System
IMU	Inertial Measurement Unit
INS	Inertial Navigation System
IoV	Internet of Vehicles
IPFS	Inter-Planetary File System
ITS	Intelligent Transportation Systems
ITL	Incremental Tracking Loss
JSON	JavaScript Object Notation
kNN	k-Nearest Neighbors
KPCA	Kernel Principal Component Analysis
LDGV	Light-Duty Gasoline Vehicle
LightGBM	Light Gradient-Boosting Machine
LR	Logistic Regression
LSTM	Long Short-Term Memory
MaaS	Mobility-as-a-Service
MAE	Mean Absolute Error
MAP	Manifold Absolute Pressure
MAPE	Mean Absolute Percentage Error
MAF	Mass Air Flow
MIL	Malfunction Indicator Lamp
ML	Machine Learning
MLP	Multi-Layer Perceptron
MLR	Multiple Linear Regression
mRMR	Maximum Relevance Minimum Redundancy
MRAE	Mean Relative Absolute Error
MSE	Mean Squared Error
MQTT	Message Queuing Telemetry Transport
MTST	Modified Time Series Transformer
NMAE	Normalized Mean Absolute Error
NNMF	Non-Negative Matrix Factorization
OBD	On-Board Diagnostics
OD	Origin–Destination
OS	Operating System
OTA	Over-the-Air
PA-LSTM	Parallel Attention-Based Long Short-Term Memory
PASER	Pavement Surface Evaluation and Rating
PCA	Principal Component Analysis
PCM	Powertrain Control Module
PEMS	Portable Emission Measurement System
PHEV	Plug-in Hybrid Electric Vehicle
PID	Parameter ID
PIE	Pedestrian Intention Estimation
PN	Particulate Number
PPCA	Probabilistic Principal Component Analysis
PSO	Particle Swarm Optimization
PTW	Powered Two-Wheeler
PVT	Probe Vehicle Trajectory
R2C	Regression-to-Classification
RBF	Radial Basis Function
RDC	Real-World Driving Cycle
RDE	Real Driving Emissions
RDR	Remaining Driving Range
REMVT	Remote Emission Management Vehicle Terminal
REP	Reduced Error Pruning
RF	Random Forest
RL	Reinforcement Learning
RLS	Recursive Least Squares
RMSE	Root Mean Square Error
RNN	Recurrent Neural Network
ROC	Receiver Operating Characteristic
ROS	Remote Operating System
RPM	Revolutions Per Minute
SatNav	Satellite Navigation
SCR	Selective Catalytic Reduction
Seq2Seq	Sequence-to-Sequence
SGD	Stochastic Gradient Descent
SMAPE	Symmetric Mean Absolute Percentage Error
SMOTE	Synthetic Minority Oversampling Technique
SMPC	Secure Multi-Party Computation
SNR	Signal-to-Noise Ratio
SoC	State of Charge
SUMO	Simulation of Urban Mobility
SUV	Sport Utility Vehicle
SVR	Support Vector Regression
SVM	Support Vector Machine
TAQ	Traffic–Air Quality
TEDA	Typicality and Eccentricity Data Analytics
TFT	Transmission Fluid Temperature
THC	Total Hydrocarbon
TinyML	Tiny Machine Learning
TNN	Transformer Neural Network
TPMS	Tire Pressure Monitoring
TPS	Throttle Position Sensor
TSS	Transmission Input/Output Shaft Speed
UDS	Unified Diagnostic Services
UHDV	Ultra-Heavy-Duty Vehicle
USB	Universal Serial Bus
V2V	Vehicle-to-Vehicle
VSP	Vehicle-Specific Power
VSS	Vehicle Speed Sensor
VT-Micro	Virginia Tech Microscopic
Wi-Fi	Wireless Fidelity
WSS	Wheel Speed Sensor
XAI	Explainable Artificial Intelligence
XGBoost	Extreme Gradient Boosting
ZEV	Zero-Emission Vehicle
